# Three Oxidative Stress-Related Genes That Associate Endometrial Immune Cells Are Considered as Potential Biomarkers for the Prediction of Unexplained Recurrent Implantation Failure

**DOI:** 10.3389/fimmu.2022.902268

**Published:** 2022-06-03

**Authors:** Jia-zhe Lin, Nuan Lin

**Affiliations:** ^1^ Neurosurgical Department, The First Affiliated Hospital of Shantou University Medical College, Shantou, China; ^2^ Obstetrics and Gynecology Department, The First Affiliated Hospital of Shantou University Medical College, Shantou, China

**Keywords:** recurrent implantation failure, oxidative stress-related gene, biomarker, endometrial immune cell, computational biology

## Abstract

Recurrent implantation failure (RIF) represents a new challenge in the field of assisted reproductive technology (ART). Considering the known effects of immune cell regulation on embryo implantation process, as well as our gene set variation analysis (GSVA) results that suggested the association between RIF and pathways of oxidative stress and immune responses, we hypothesized that oxidative stress- related genes (OSGs) associated with aberrant immunological factor may represent novel biomarkers for unexplained RIF. We therefore screened out the immune cell coexpressed OSGs by performing CIBERSORT, LM22 matrix and Pearson correlation, followed by constructing an OSG signature by least absolute shrinkage and selection operator (LASSO) regression. Three OSGs (AXL, SLC7A11 and UBQLN1) were then identified to establish a RIF risk signature, which showed high ability to discriminating RIF from fertile control. A nomogram was established, with a free online calculator for easier clinical application. Finally, Chilibot, protein-protein interaction analysis and BioGPS were sequentially applied for the investigation of functional relationships of these three genes with RIF and other OSGs, as well as their expression abundance across different human tissues. In conclusion, we identified an OSG signature that are relevant novel markers for the occurrence of unexplained RIF.

## Introduction

The breakthrough of assisted reproductive technology (ART) in recent years has improved outcomes for millions of struggling couples who fail to conceive. However, recurrent implantation failure (RIF), generally defined as the failure of implantation after more than three replacement cycles of high quality embryos or after transferring a total of more than ten embryos, represents a new challenge in the field of ART and leave couples frustrated (1). The diagnosis of RIF is a difficult reality for both couples and clinical practitioners, since it has led to the waste of precious embryos while treatments are always empirical with limited efficacy (2). Thus, it would help to establish a set of standardized tests for the prediction of RIF occurrence as a preliminary evaluation on each patient before embryo transfer, which would then hopefully direct the personal approach for each individual and prevent the waste of embryos.

RIF is a complex problem with a wide variety of etiologies and mechanisms. However, for unexplained RIF, in which no other conditions are known to be the cause, the endometrial immune dysregulation has been regarded as one of the most important factors. The decidualized endometrial stromal cells are able to suppress the local maternal immune response, which are the prerequisites for protecting embryos from immunological attacks (3), and thus critical to implantation (4). While there are emerging investigation into the endometrial immune profile in RIF, however, translation to clinical practice is limited due to the lack of predictive biomarkers.

Oxidative stress (OS), a state resulted from the imbalanced state between pro-oxidant molecules and antioxidant defense levels, has been recognized to play a key role of a variety of female reproductive disorders, including the occurrence of spontaneous and recurrent miscarriage by negatively affecting embryo implantation (5, 6). Shahin et al. also reported that low-level microwave irradiation may lead to implantation failure by inducing OS in mice (7). Considering the important role of OS-mediated regulation during embryo implantation, it is thus meaningful to search for key OS related biomarkers capable of identifying individuals who are at risk of RIF.

Based on the preliminary results that RIF is associated both with OS and immune dysregulation of the endometrium in this study, we therefore aim to identify the key oxidative stress- related genes (OSGs) of predictive significance for the occurrence of unexplained RIF. Intriguingly, we discovered that that the constructed OS related gene risk signature, which is closely associated with endometrial immune cells, can accurately predicts RIF occurrence. Given the widely accepted consensus of endometrial immune dysregulation as an important contributor to unexplained RIF and the emerging role of OS in the context of embryo implantation, our findings indicate potential clinical implications for this OS related gene signature as predictive biomarkers as well as therapeutic target to improve pregnancy outcomes among patients undergoing ART procedure.

## Materials and Methods

### Clinical Data Collection and Processing

The RNA sequencing (RNA-seq) expression profiles were downloaded from GEO database (2020.5, https://www.ncbi.nlm.nih.gov/geo/). Data were extracted from GSE111974 providing endometrial RNA expression from patients with RIF and fertile controls. By using data from Ensemble (https://uswest.ensembl.org/index.html) (8), we re-annotated the gene symbols and extracted mRNAs from the original dataset. The expression profiles, including mRNA expressions from 24 normal endometrial tissues and 24 RIF endometrial tissue, were presented by normalized signal intensity. Finally, 14944 mRNAs from 48 samples were extracted.

Principal component analysis (PCA) is a method for reducing the dimensionality of large datasets, increasing interpretability but at the same time minimizing information loss (9). Thus, PCA was then employed to find out the distribution of these mRNAs between RIF and fertile control graphically.

### Functional Enrichment Analysis of RIF Associated Genes

Gene Set Variation Analysis (GSVA, https://www.broadinstitute.org/gsea/index.jsp) was applied to assess the underlying changes in pathway activities and biological functions between RIF and fertile controls. GSVA is a non-parametric unsupervised method that transforms the genes of the sample matrix into predefined gene sets without *a priori* knowledge of experiment design (10). In this study, we used the R package “GSVA” to calculate the score of each patient based on previously defined gene sets of Gene Ontology (GO) and Kyoto Encyclopedia of Genes and Genomes (KEGG) pathways. The gene sets “c5.all.v7.1.symbols.gmt” and “c2.cp.kegg.v6.2.symbols.gmt”, downloaded from the Molecular Signature Database (MSigDB, http://software.broadinstitute.org/gsea/msigdb/index.jsp), were selected and combined as the reference. Subsequently, the R package “limma” was used to build linear models for comparing the GSVA scores between RIF patients and fertile controls. Pathways with adjusted P value < 0.25 and | log2FC| ≥ 0.1 were considered as significantly altered.

### Filtering of OSGs

OSGs were extracted from the GO_ OXIDATIVE_STRESS gene set in Molecular Signatures Database v7.1 website (https://www.gsea-msigdb.org/gsea/msigdb/index.jsp). The mRNAs levels of these OSG were filtered for further analysis.

### Immune Cell Composition Analysis

Based on the gene expression profile of a sample, CIBERSORT enables the characterization of the cellular composition of complex tissues, such as solid tumor. This robust and novel method has been shown to perform better than other methods when dealing with background noises, unknown contents and closely associated cell types (11). LM22, a leukocyte gene signature matrix containing a total of 547 genes, was developed and validated to distinguish 22 human hematopoietic cell subtypes, including seven T cell types, naive and memory B cells, plasma cells, natural killer (NK) cells and myeloid subsets (12). The immune cell compositions in RIF and fertile controls, were evaluated using CIBERSORT in combination with the LM22 matrix. Only cases with a P< 0.05 of CIBERSORT outputs were selected for further analysis. The correlation between the expression of OSGs and immune infiltration, as well as among different immune cell types were calculated by Pearson correlation. Sankey diagram was employed to show the relationship between the pair of OSGs and immune cell, with P value < 0.05 and | Pearson correlation coefficient | > 0.6.

### Identification of an OSG Signature for Predicting RIF

To explore if the immune-cell co-expressed OSGs could prompt the prediction of RIF, a predicting model was constructed based on the immune-cell co-expressed OSGs by applying the “glmnet” package, version 3.0-2 (https://cran.r-project.org/) in R language. Least absolute shrinkage and selection operator (LASSO) regression was performed to further select the optimal predictive features. A multivariable logistic regression model and a nomogram were constructed by integrating the features with non-zero coefficients in LASSO regression in order to prevent model overfitting. The RIF prediction model was then built according to the regression coefficient-weighted mRNA expression and a predicted score formula was established as follows:


$$\text{Predicted score = }\sum_{i=1}^{n}\text{Expi}\times \text{Coei}$$

In the formula, N, Expi, Coei indicates the number of selected mRNAs, the expression value of each mRNA and the multivariate Cox regression coefficient, respectively. Next, model sensitivity and specificity were evaluated by receiver operating characteristic (ROC) analyses. A nomogram, as the visualization of linear prediction model, was constructed based on all the significant factors for RIF prediction. In order to facilitate clinical use, a free online calculator for the final nomogram was designed by DEnorm package (version 5.0.1, https://cran.r-project.org/) and published in “https://www.shinyapps.io/”.

### Chilibot and Protein-Protein Interaction Analysis

Chilibot analysis was then performed (http://www.chilibot.net) (13). Within the range of user-defined terms, Chilibot determined those already known relationships by searching for their co-occurrence in the same sentence across the abstracts of Pubmed. As a result, much closer relations can be identified by Chilibot analysis compared to a plain Pubmed search, followed by the association of the same sentence coexistence to a stimulatory, or inhibitory, or noninteractive relationship (14). Based on the above principle, a “two-list analysis” was performed, with the first list containing the term “Recurrent Implantation Failure” and the second list containing OSG list. In addition, protein-protein interaction (PPI) analysis was employed to demonstrate the functional networks of OSGs. By collecting, scoring and integrating all publicly available sources, the Search Tool for the Retrieval of Interacting Genes (STRING, version 11.5, http://string-db.org) database provides references for studying the mechanism of disease occurrence or progression and provides a basis for exploring the functional PPI (15). Cytoscape 3.5.0 (version 3.7.2; https://cytoscape.org/) was used to construct and visualize the results from the PPI network, with the criteria set as a combined score of > 0.15.

### Visualization of Core Gene Expressions Across Different Human Tissues

Providing comprehensive resources about gene and protein function, the BioGPS (http://biogps.org/#goto=welcome) is a free online extensible and customizable gene annotation portal tool, and thus was applied to identify the expressions of newly identified core genes across different human tissues.

### Statistical Analysis

Quantitative data were presented as the mean ± standard deviation. Statistical differences between two groups were examined by the Wilcoxon test. All statistical tests were conducted using R programming language. P < 0.05 was considered statistically significant.

## Results

### Identification of OSGs Associated Pathways *via* GSVA

**Figure 1:**
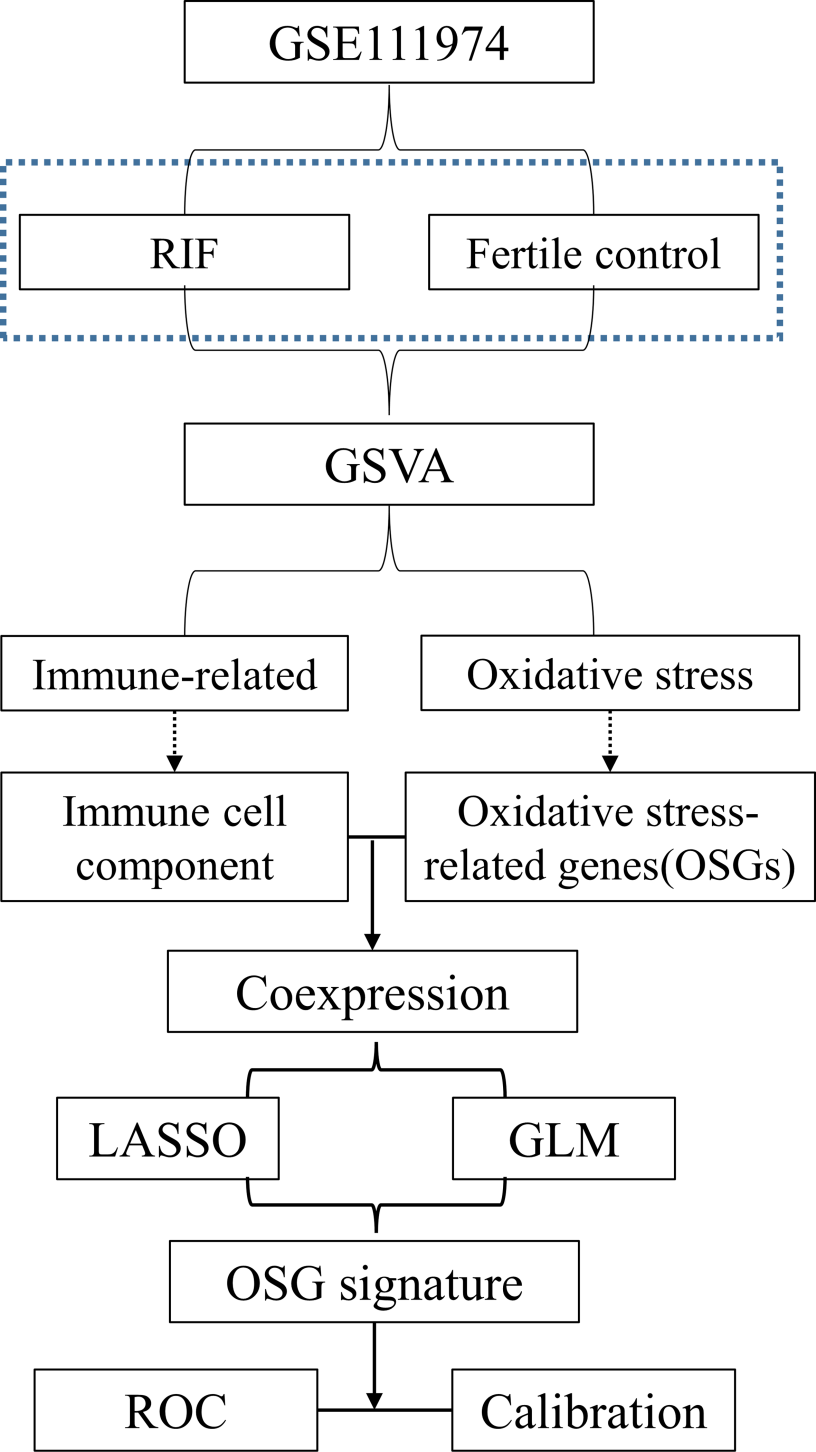
Flow chart of data preparation, processing, analysis.

**Figure 2:**
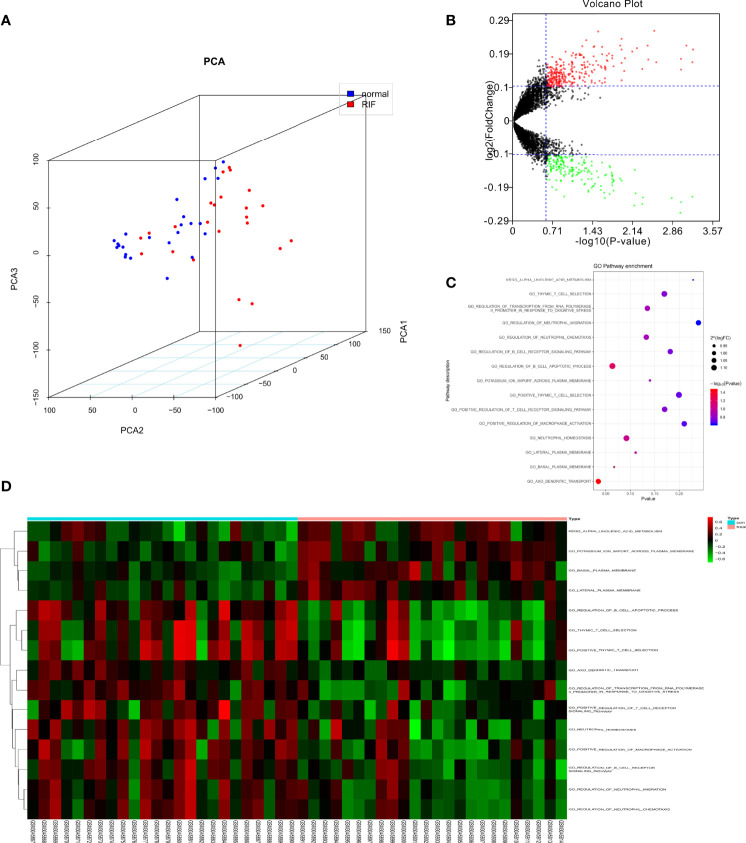
GSVA analysis displaying immune related pathway differences. **(A)** Principal components analysis of lncRNAs between normal and recurrent implantation failure (RIF) sample. **(B)** Volcano plot showed the differences in pathway activities scored by GSVA between normal and RIF sample. Differences in pathway activities scored by GSVA between normal and RIF sample. **(C)** Bubble plot showed the analysis result. **(D)** Heatmap exhibited the differences of the pathway.

A workflow of this study was shown in **Figure 1**. PCA was employed to figure out the distribution of the endometrial mRNAs from RIF and fertile control graphically (**Figure 2A**). As **Figure 2A** shown, the RNA expression from all samples clearly shows two different distributed clusters, from RIF patients and fertile control respectively, suggesting that there may be significant differences between RIF patients and the fertile controls in terms of RNA expression level and biological function. To reflect the functional characteristics of RIF endometrial tissue, GSVA was employed and 475 pathways were significantly activated in RIF according to GSVA results (**Table S1**), with results shown as volcano plot (**Figure 2B**). Among these pathways, there are 15 signaling found to be related to immune response and oxidative stress reaction (**Figures 2C, D**), indicating that immune response and oxidative stress regulation may be differentially activated in the endometrium of between RIF patients and the fertile controls. We thus ask if there are certain OSGs that regulate immune cells can serve as predictive biomarkers for the occurrence of RIF.

### Identification of Immune Cell Landscapes of RIF and Immune Cell Regulated OSG

**Figure 3:**
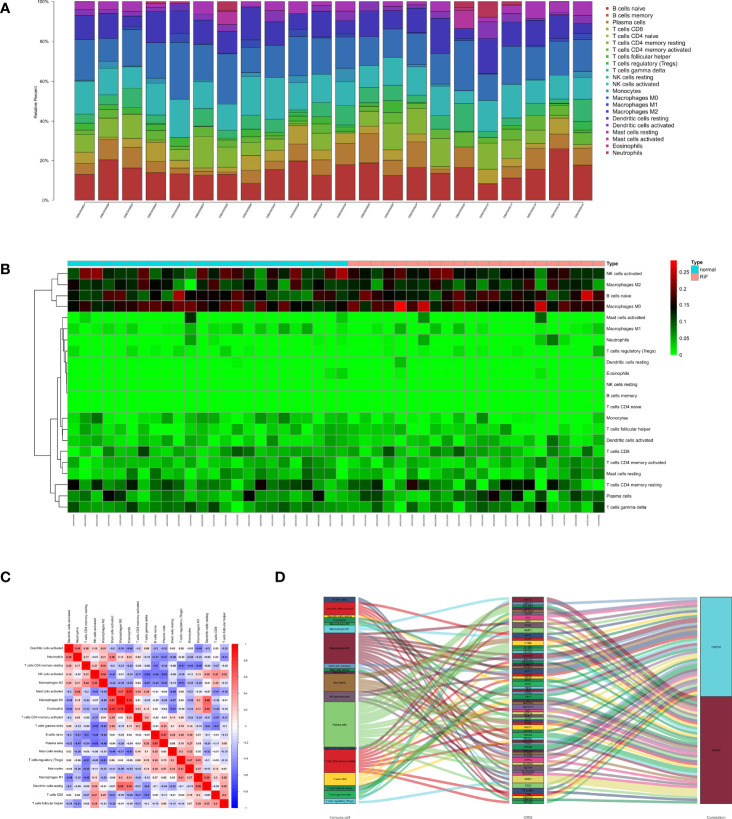
Distribution of immune cells between normal and recurrent implantation failure sample using CIBERSORT for all eligible samples in the derivation cohort. **(A)** Barplot showed the fractions of immune cells in individual samples. **(B)** Heatmap showed normalized absolute abundance for each cell type in individual samples. **(C)** Correlation heatmap showed gene co-expression patterns among significantly altered immune cells. **(D)** Sankey diagram showed the relationships between the immune cells and their co-expressed OSGs.

To investigate the immune landscapes and characterize the cell compositions of RIF, CIBERSORT in combination with the LM22 matrix was employed. The distribution of 22 immune cell types in RIF was shown in a barplot (**Figure 3A**) and heatmap (**Figure 3B**), indicating that many of them were significantly altered. To further identify the co-expression patterns among these significantly altered immune cells, we performed Pearson correlation analysis to evaluate their potential underlying relationships and discovered that there were extensive correlations among the content of different immune cell, suggesting that immune cells are mostly altered as a whole in RIF (**Figure 3C**). Meanwhile, a total of 453 OSG were extracted from the GO_ OXIDATIVE_STRESS gene set in Molecular Signatures Database (**Table S2**). We next analyzed the correlation between the immune related OSGs and the infiltration of immune cell subtypes in RIF. The correlation values of immune cells and the OSGs were shown in **Figure 3D**. The 8 OSGs (TP53INP1, FABP1, BNIP3, AXL, SLC7A11, ENDOG, UBQLN1, and NCF1) that are immune-cell correlated with P value < 0.05 and | Pearson correlation coefficient | > 0.7 were selected.

### Construction of the OSG Signature for RIF Prediction

**Figure 4:**
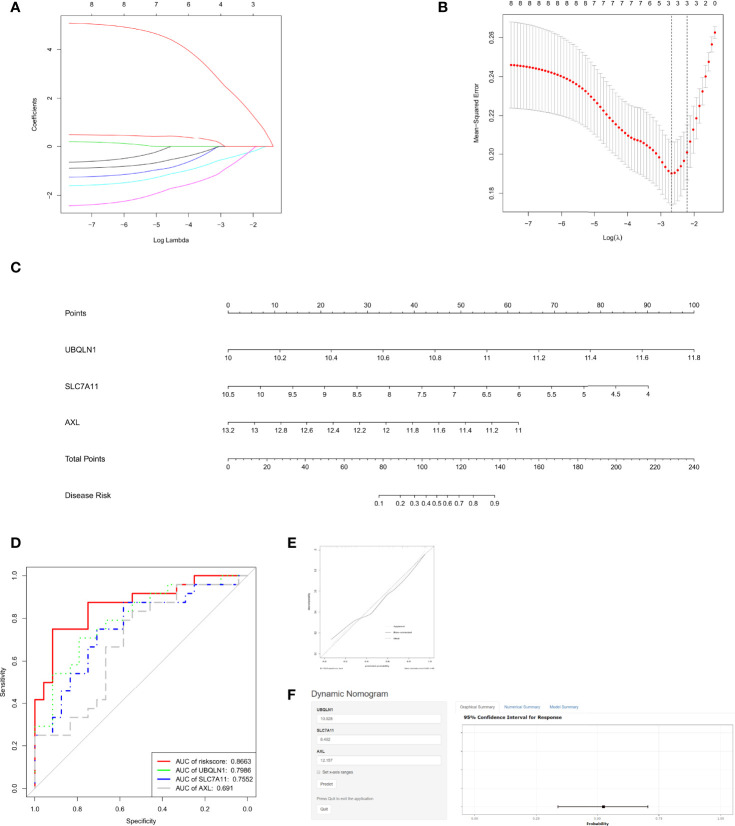
Assessment of the recurrent implantation failure (RIF) predictive value of OSGs. **(A)** Log (Lambda) value of OSG. in least absolute shrinkage and selection operator (LASSO) model. **(B)** The most appropriate log (Lambda) value in the LASSO model. **(C)** Nomogram was developed by integrating the risk signature. **(D)** Receiver operating characteristic (ROC) curves for the RIF prediction according to the nomogram and each gene expression. **(E)** The calibration curve of the model. **(F)** The user-friendly free online calculator to facilitate the use of the nomogram.

To further select immune cell regulated OSGs that prompt the prediction of RIF, LASSO regression was used to select appropriate parameters by determining interpretable prediction rules in high dimension data. Three mRNAs, as AXL, SLC7A11 and UBQLN1, were identified (**Figures 4A, B**). Subsequently, the predicted score formula was constructed by generalized linear model and shown as follow:

Risk score= (-2.4140 × expression level of AXL) + (-1.1539 × expression level of SLC7A11) + (3.5932 × expression level of UBQLN1)

Pearson analysis further confirm strong correlations (all >0.7) between AXL and T cells, SLC7A11 and NK cells, UBQLN1 and dendritic cells (**Table 1**).

**Table 1:** Correlations between critical OSGs and immune cells.

Immune cell	Gene	P value	Correlation coefficient
T cells gamma delta	AXL	<0.001	-0.79164
NK cells activated	SLC7A11	<0.001	0.774499
Dendritic cells activated	UBQLN1	<0.001	-0.70291

To make it easier for application, AXL, SLC7A11 and UBQLN1 levels were incorporated into a multivariable logistic regression model to build a predictive model for RIF and presented as a nomogram (**Figure 4C**). On the nomogram graph, each mRNA expression level represents a score, and the total score maps the RIF probability.

We next drew the ROC curves (**Figure 4D**) and calibration curves (**Figure 4E**) for the OSG signature. **Figure 4D** reports the corresponding ROC curve for the expression values: AXL, SLC7A11, UBQLN1 and the 3- OSG risk score, shows an AUC of 0.691, 0.7552, 0.7986 and 0.8663, respectively. The curves clearly showed that all of the three genes relatively high sensitivity and specificity values, but the signature based on them had the best predictive value (AUC=0.8663) in distinguishing RIF patients from fertile controls.

Calibration plot showed the mean predicted probability of outcome against the observed proportion of outcomes for 48 groups based on the calculated RIF risk score. A locally weighted regression, indicated by dashed line, was plotted to demonstrate the general trend and the ideal result. Using a bootstrap approach, the bias-corrected line was produced to estimate predicted and observed values based on a nonparametric smoother applied to a sequence of predicted values. Overall, the apparent and bias-corrected lines were well aligned and shifted slightly from the ideal 45-degree line, indicating good predictive performance. The bias- corrected line and apparent line crossed with the ideal line at around 35% of the predicted value. When the predicted risk is less than 35%, these two lines were above the ideal line, indicating that the predicted risk will be a bit overestimated. In contrast, when the predicted risk is greater than 35%, the two lines are both below the ideal line, suggesting that the risk will be a bit underestimated.

In order to facilitate clinical application, an online app based on the nomogram was established to distinguish RIF patients from fertile controls (**Figure 4E**). By simply inputting the endometrial mRNA expression levels of AXL, SLC7A11and UBQLN1 of a patient, clinicians who adopt the nomogram can quickly calculate the patient’s probability of RIF occurrence, as well as checking the confidence interval of the predicted results in the online app (**Figure 4F**): https://linnuanstu2009.shinyapps.io/dynnomapp/?_ga=2.24486970.1046640858.1591781847-159983587.1588641469.

### Chilibot and Protein-Protein Interaction Analysis

**Figure 5:**
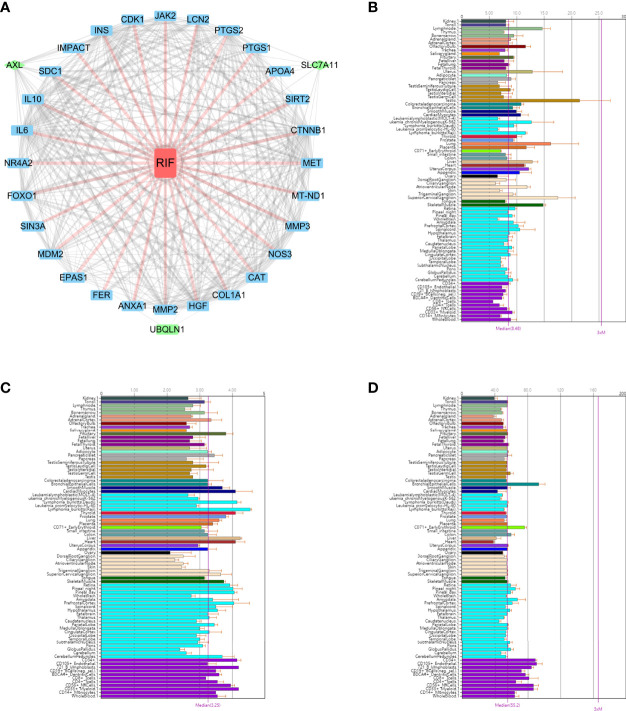
The functional annotation of the three key OSGs. **(A)** PPI analysis revealed the correlation among the 3 genes and 28 OSGs. BioGPS showed the expression level of **(B)** AXL, **(C)** SLC7A11 and **(D)** UBQLN1 on normal human tissues by BioGPS.

The molecular mechanisms underlying the functional interaction of RIF with OSGs, including the screened out AXL, SLC7A11and UBQLN1, were investigated with Chilibot analysis. According to Chilibot analysis, no direct and strong relationship between RIF with these three genes has been reported so far, while there are 28 OSGs previously found to be associated with RIF. In addition, PPI analysis revealed the correlation among the 3 genes and 28 OSGs (**Figure 5A**), suggesting that AXL, SLC7A11and UBQLN1 as potential RIF markers is innovative and their interaction to RIF may be mediated *via* molecular pathways involving other OSGs.

### Comparison of the Expression Level of Core Genes on Normal Human Tissues by BioGPS

We used BioGPS to identify the hub gene expression in normal tissues. This data set demonstrated a survey across various normal human tissues (platform: U133plus2 Affymetrix microarray) (16). The above mentioned three key biomarkers were imported in the BioGps database for organ positioning. The results showed that AXL (**Figure 5B**), SLC7A11 (**Figure 5C**) and UBQLN1 (**Figure 5D**) were all moderately abundant in uterus, suggesting the feasibility of detecting these three genes in endometrium as biomarkers for RIF prediction in ART practice.

## Discussion

During embryo implantation, a localized uterine tissue reaction called decidualization, profoundly influences the differentiation and trafficking of several leukocyte subtypes. Thus, aberrant immunological factors has been generally believed as an important source of the occurrence of recurrent miscarriage and implantation failure, evidenced by the fact that there is local immune overactivation in the endometrium in 56.6% of the patients who experienced unexplained RIF (17). Liu et al. found that the dysregulation of uterine immune activity and the exacerbation of oxidative stress can lead to a reduced implantation sites (18). Consistently in this study, our GSEA result based on the endometrial RNAseq of 24 RIF and 24 fertile women showed that the occurrence of unexplained RIF is correlated to both OS and immune dysregulation. We thus ask if the OSGs that are associated with immune cells can serve as predictive biomarkers for the occurrence of unexplained RIF. Using LASSO regression, a prediction model for RIF based on three OSGs (UBQLN1, SLC7A11 and AXL) is constructed with good differential performance, and potentially underlie the pathophysiology of RIF with association of immune cells activities, especially dendritic cells, NK cells and T lymphocytes.

The SLC7A11 (Solute Carrier Family 7 Member 11) gene belongs to the solute transport family and encodes a heteromeric, sodium-independent and highly specific cystine/glutamate xCT transporter, which is believed to be involved in regulating ferroptosis in the diseases characterized by iron deposition and reactive oxygen species production, such as ischemia/reperfusion-induced acute lung injury and cardiomyopathies (19–21). It has been reported that the expression of SLC7A11 is promoted to enhance cell resistance in intestines when cells are under oxidative stress (19). Given that a recent endometrial proteome approach during the window of implantation reveals that the pathway of ferroptosis is altered in RIF patients (22), the ferroptosis- related SLC7A11 gene may be critically involved in the pathogenesis of RIF. AXL (AXL Receptor Tyrosine Kinase) encodes a protein member of the Tyro3-Axl-Mer (TAM) receptor tyrosine kinase subfamily. The molecular system of AXL and its ligand GAS6, is traditionally considered as a negative regulator of the immune response and participate in the development and progression of a variety of inflammatory diseases (23–25). Consistently, we found that AXL is a protective factor in the development of RIF. In addition, the induction of OS by Gas6/AXL signaling was recently found to be placenta- specific while no OS changes were observed in the other maternal rat organs (26), suggesting that AXL may be critically involved in the immune responses at the feto-maternal interface by regulating OS. UBQLN1(Ubiquilin 1) encodes an ubiquitin-like protein (ubiquilin) and plays important roles in the regulation of oxidative stress-induced apoptotic signaling pathway (27), as well as a variety of protein degradation mechanism and pathways including ubiquitin-proteasome system (UPS) and autophagy, which is found to be activated during murine and human endometrial stromal cell decidualization (28). Thus, UBQLN1 may serve as a potential critical gene underlying RIF. Meanwhile, these three genes are coexpressed with several certain subtypes of immune cells including dendritic cells, NK cells and T lymphocytes, all of which have been widely believed to play important roles during the process of implantation. Human uterine natural killer (uNK) cells are the most abundant lymphocyte population at the onset of decidualization, constituting 70% of total uterine leukocytes (29). Adequate activated uNK cells mainly produce immunoregulatory and angiogenic cytokines, which are important for maternal tolerance during the implantation window (30, 31). Similarly, a failure of maternal dendritic cells (DC) to monitor the maternal/fetal interface has major implications for afferent fetomaternal tolerance mechanisms (32). These specialized antigen-presenting cells are capable of either inducing an adaptive immune response followed by fetal loss or promoting immune tolerance at the fetomaternal interface during the early phase of human pregnancy (33–35). A local T helper cell homeostasis has been observed during the implantation window and is fundamental to the establishment of maternal tolerance (17, 36). The immune switch, from a type 1 T helper (Th1) pro-inflammatory environment to a type 2 T helper (Th2) anti- inflammatory environment, allows development of local mechanisms promoting immunotrophism, as well as down-regulation of inflammation and cytotoxic pathways (37).

A previous study constructed a 303-gene signature for predicting RIF. However, to the best of our knowledge, we are the first to identify an OSG risk signature with correlation to endometrial immune cells. This finding indicates the aberrant immunological factors, which have been widely considered as source of unexplained RIF, may potentially interrupt embryo implantation associated with OSG regulation. The genes as well as the signature underlying oxidative stress and immune response, may serve as potential novel markers for RIF.

In conclusion, the present study highlights 3 genes (SLC7A11, AXL and UBQLN1), known to be associated with both oxidative stress and immune cells in the endometrium, as novel relevant markers for RIF. The risk signature built based on these three genes represents valuable novel prediction model of RIF occurrence.

## Data Availability Statement

Publicly available datasets were analyzed in this study. This data can be found here: https://www.ncbi.nlm.nih.gov/geo/query/acc.cgi?acc=GSE111974 Gene Expression Omnibus (GEO) GSE111974.

## Author Contributions

Software, formal analysis, data curation, review and editing: J-zL. Conceptualization, methodology, writing, funding acquisition: NL; All authors have read and agreed to the published version of the manuscript.

## Funding

The present study was supported by Guangdong Science and Technology Special Fund (grant no. [2021] 88-40).

## Conflict of Interest

The authors declare that the research was conducted in the absence of any commercial or financial relationships that could be construed as a potential conflict of interest.

## Publisher’s Note

All claims expressed in this article are solely those of the authors and do not necessarily represent those of their affiliated organizations, or those of the publisher, the editors and the reviewers. Any product that may be evaluated in this article, or claim that may be made by its manufacturer, is not guaranteed or endorsed by the publisher.
