# Supplementary material for: Three Oxidative Stress-Related Genes That Associate Endometrial Immune Cells Are Considered as Potential Biomarkers for the Prediction of Unexplained Recurrent Implantation Failure
Source: Front Immunol. 2022 Jun 3;13:902268. doi: 10.3389/fimmu.2022.902268 (PMC9203891; doi:10.3389/fimmu.2022.902268)
Supplement: Supplementary file 1 [file Table_1.docx]

Table S1 475 pathways were signiﬁcantly activated in RIF

| id | logFC | AveExpr | t | P.Value | adj.P.Val | B |
| --- | --- | --- | --- | --- | --- | --- |
| GO_PROTEIN_NEDDYLATION | 0.25604 | -0.00061 | 6.309376 | 6.46E-08 | 0.000267 | 7.928249 |
| GO_SYNAPTIC_VESICLE_TRANSPORT | 0.176001 | 0.00755 | 6.221087 | 8.91E-08 | 0.000267 | 7.636259 |
| GO_PIGMENT_GRANULE_ORGANIZATION | 0.207051 | -0.00263 | 5.879225 | 3.08E-07 | 0.000615 | 6.508928 |
| GO_RAL_GTPASE_BINDING | -0.2169 | 0.005913 | -5.74942 | 4.91E-07 | 0.000646 | 6.082848 |
| GO_SYNAPTIC_VESICLE_LOCALIZATION | 0.171863 | 0.011114 | 5.723563 | 5.39E-07 | 0.000646 | 5.998158 |
| GO_SYNAPTIC_VESICLE_CYTOSKELETAL_TRANSPORT | 0.219573 | 0.012849 | 5.609486 | 8.12E-07 | 0.000811 | 5.625214 |
| GO_AXON_CYTOPLASM | 0.150666 | 0.007322 | 5.472041 | 1.33E-06 | 0.001022 | 5.177704 |
| GO_NEGATIVE_REGULATION_OF_SIGNAL_TRANSDUCTION_BY_P53_CLASS_MEDIATOR | 0.180483 | 0.012081 | 5.454967 | 1.41E-06 | 0.001022 | 5.122269 |
| GO_INHIBITORY_POSTSYNAPTIC_POTENTIAL | -0.23253 | -0.00451 | -5.43116 | 1.53E-06 | 0.001022 | 5.045018 |
| GO_POSITIVE_REGULATION_OF_VASCULAR_ASSOCIATED_SMOOTH_MUSCLE_CELL_MIGRATION | -0.26404 | -0.01568 | -5.39672 | 1.73E-06 | 0.00104 | 4.933426 |
| GO_REGULATION_OF_SKELETAL_MUSCLE_ADAPTATION | -0.22232 | -0.00744 | -5.26544 | 2.76E-06 | 0.001506 | 4.509493 |
| GO_NEGATIVE_REGULATION_OF_INTRINSIC_APOPTOTIC_SIGNALING_PATHWAY_IN_RESPONSE_TO_DNA_DAMAGE | 0.171017 | 0.006733 | 5.061403 | 5.66E-06 | 0.002829 | 3.855884 |
| GO_PROTEASOME_ASSEMBLY | 0.261151 | 0.018985 | 5.013923 | 6.69E-06 | 0.003083 | 3.704814 |
| GO_PIGMENT_ACCUMULATION | 0.178537 | 0.007203 | 4.92251 | 9.19E-06 | 0.003935 | 3.415162 |
| GO_UBIQUITIN_PROTEIN_TRANSFERASE_REGULATOR_ACTIVITY | 0.219655 | 0.016816 | 4.895606 | 1.01E-05 | 0.004032 | 3.330227 |
| GO_CAMP_RESPONSE_ELEMENT_BINDING | -0.21943 | -0.00575 | -4.82125 | 1.30E-05 | 0.004889 | 3.096276 |
| GO_REGULATION_OF_INTRINSIC_APOPTOTIC_SIGNALING_PATHWAY_IN_RESPONSE_TO_DNA_DAMAGE | 0.152209 | 0.005413 | 4.788204 | 1.46E-05 | 0.004962 | 2.992672 |
| GO_TYPE_B_PANCREATIC_CELL_APOPTOTIC_PROCESS | -0.23853 | 0.010358 | -4.78275 | 1.49E-05 | 0.004962 | 2.975612 |
| GO_TRANSLATION_PREINITIATION_COMPLEX | 0.212515 | 0.007912 | 4.705599 | 1.94E-05 | 0.006126 | 2.73478 |
| GO_REGULATION_OF_RETINOIC_ACID_RECEPTOR_SIGNALING_PATHWAY | -0.20847 | 0.004556 | -4.67459 | 2.16E-05 | 0.006471 | 2.638368 |
| GO_PHOSPHATASE_INHIBITOR_ACTIVITY | -0.1402 | -0.00198 | -4.64711 | 2.37E-05 | 0.006769 | 2.553151 |
| GO_NEGATIVE_REGULATION_OF_ASTROCYTE_DIFFERENTIATION | -0.24261 | -0.02539 | -4.62913 | 2.52E-05 | 0.00687 | 2.497479 |
| GO_CARBON_SULFUR_LYASE_ACTIVITY | 0.202135 | 0.014305 | 4.587408 | 2.91E-05 | 0.007572 | 2.368602 |
| GO_DISULFIDE_OXIDOREDUCTASE_ACTIVITY | 0.182557 | 0.016314 | 4.490596 | 4.03E-05 | 0.010068 | 2.071295 |
| GO_ANTEROGRADE_AXONAL_TRANSPORT | 0.145441 | 0.004231 | 4.470146 | 4.32E-05 | 0.010354 | 2.008814 |
| GO_EPITHELIAL_CELL_MATURATION | -0.17103 | 0.001675 | -4.4247 | 5.03E-05 | 0.011597 | 1.870386 |
| GO_NEGATIVE_REGULATION_OF_MITOCHONDRION_ORGANIZATION | 0.118034 | 0.007426 | 4.396143 | 5.53E-05 | 0.011657 | 1.783679 |
| GO_REGULATION_OF_CELLULAR_AMINO_ACID_METABOLIC_PROCESS | 0.206933 | 0.027133 | 4.393454 | 5.58E-05 | 0.011657 | 1.775528 |
| GO_CYCLIC_NUCLEOTIDE_PHOSPHODIESTERASE_ACTIVITY | 0.169797 | 0.013121 | 4.384709 | 5.75E-05 | 0.011657 | 1.749032 |
| GO_GEMINI_OF_COILED_BODIES | 0.238184 | 0.013126 | 4.380394 | 5.83E-05 | 0.011657 | 1.735968 |
| GO_CELLULAR_RESPONSE_TO_ATP | -0.16492 | 0.004852 | -4.34476 | 6.57E-05 | 0.012351 | 1.628279 |
| GO_POSITIVE_REGULATION_OF_TRANSCRIPTION_INITIATION_FROM_RNA_POLYMERASE_II_PROMOTER | 0.219888 | 0.006804 | 4.343678 | 6.59E-05 | 0.012351 | 1.625015 |
| GO_K63_LINKED_POLYUBIQUITIN_MODIFICATION_DEPENDENT_PROTEIN_BINDING | -0.17319 | 0.000534 | -4.25244 | 8.92E-05 | 0.0162 | 1.351068 |
| GO_NEGATIVE_REGULATION_OF_INTRINSIC_APOPTOTIC_SIGNALING_PATHWAY_BY_P53_CLASS_MEDIATOR | 0.160086 | 0.01346 | 4.243508 | 9.19E-05 | 0.0162 | 1.32437 |
| GO_TAU_PROTEIN_KINASE_ACTIVITY | -0.16937 | 0.009236 | -4.22739 | 9.69E-05 | 0.016317 | 1.276271 |
| GO_TELENCEPHALON_GLIAL_CELL_MIGRATION | -0.20082 | -0.00694 | -4.21682 | 0.0001 | 0.016317 | 1.244804 |
| GO_CEREBRAL_CORTEX_RADIALLY_ORIENTED_CELL_MIGRATION | -0.17143 | -0.00172 | -4.21565 | 0.000101 | 0.016317 | 1.241322 |
| GO_LOCOMOTOR_RHYTHM | -0.15411 | 0.004086 | -4.19634 | 0.000107 | 0.016929 | 1.183877 |
| GO_NEGATIVE_REGULATION_OF_GLIOGENESIS | -0.15719 | -0.00715 | -4.17995 | 0.000113 | 0.016965 | 1.135242 |
| GO_NEGATIVE_REGULATION_OF_NECROTIC_CELL_DEATH | -0.18524 | -0.00078 | -4.16798 | 0.000118 | 0.016965 | 1.099747 |
| GO_REGULATION_OF_DNA_METHYLATION | -0.17677 | -0.0072 | -4.16514 | 0.000119 | 0.016965 | 1.091342 |
| GO_NEGATIVE_REGULATION_OF_GLIAL_CELL_DIFFERENTIATION | -0.16509 | -0.01437 | -4.15254 | 0.000124 | 0.016965 | 1.054084 |
| GO_RIBONUCLEOSIDE_CATABOLIC_PROCESS | 0.183167 | 0.017475 | 4.150842 | 0.000125 | 0.016965 | 1.049052 |
| GO_REGULATION_OF_RNA_POLYMERASE_II_TRANSCRIPTIONAL_PREINITIATION_COMPLEX_ASSEMBLY | 0.190557 | -0.01024 | 4.14437 | 0.000127 | 0.016965 | 1.029928 |
| GO_MAMMARY_GLAND_ALVEOLUS_DEVELOPMENT | -0.15836 | -0.00987 | -4.14408 | 0.000127 | 0.016965 | 1.029061 |
| KEGG_ARRHYTHMOGENIC_RIGHT_VENTRICULAR_CARDIOMYOPATHY_ARVC | -0.14852 | -0.00219 | -4.21627 | 9.64E-05 | 0.017455 | 1.263258 |
| GO_CYTOPLASMIC_PATTERN_RECOGNITION_RECEPTOR_SIGNALING_PATHWAY | -0.13201 | 0.006373 | -4.10831 | 0.000143 | 0.018649 | 0.923618 |
| GO_MICROVILLUS_MEMBRANE | -0.20925 | 0.011599 | -4.09197 | 0.000151 | 0.019249 | 0.875595 |
| GO_T_TUBULE | -0.12922 | -0.00336 | -4.08517 | 0.000154 | 0.019269 | 0.85564 |
| GO_DEOXYRIBONUCLEOSIDE_TRIPHOSPHATE_METABOLIC_PROCESS | 0.196163 | 0.014495 | 4.070631 | 0.000162 | 0.019787 | 0.813026 |
| GO_POSITIVE_REGULATION_OF_TUMOR_NECROSIS_FACTOR_SECRETION | 0.17448 | 0.014389 | 4.051553 | 0.000172 | 0.020628 | 0.75721 |
| GO_REGULATION_OF_INTRINSIC_APOPTOTIC_SIGNALING_PATHWAY_IN_RESPONSE_TO_DNA_DAMAGE_BY_P53_CLASS_MEDIATOR | 0.190396 | 0.007455 | 4.039357 | 0.000179 | 0.021037 | 0.721597 |
| GO_POSITIVE_REGULATION_OF_DNA_TEMPLATED_TRANSCRIPTION_INITIATION | 0.17706 | 0.009361 | 4.032238 | 0.000183 | 0.021112 | 0.700833 |
| GO_NUCLEAR_OUTER_MEMBRANE | -0.15454 | 0.004776 | -4.01271 | 0.000195 | 0.022061 | 0.643966 |
| GO_RNA_POLYMERASE_II_PREINITIATION_COMPLEX_ASSEMBLY | 0.150683 | 0.00318 | 3.99647 | 0.000206 | 0.022769 | 0.596778 |
| GO_ANTIGEN_PROCESSING_AND_PRESENTATION_OF_PEPTIDE_ANTIGEN_VIA_MHC_CLASS_I | 0.156597 | 0.013543 | 3.9914 | 0.000209 | 0.022769 | 0.582066 |
| GO_SUBSTANTIA_NIGRA_DEVELOPMENT | 0.152739 | 0.017549 | 3.980972 | 0.000216 | 0.022863 | 0.551835 |
| GO_NEURON_PROJECTION_CYTOPLASM | 0.113394 | 0.005614 | 3.979003 | 0.000217 | 0.022863 | 0.546133 |
| GO_REGULATION_OF_CELLULAR_AMINE_METABOLIC_PROCESS | 0.160252 | 0.014932 | 3.965839 | 0.000227 | 0.023312 | 0.508035 |
| GO_PROTEIN_BINDING_INVOLVED_IN_HETEROTYPIC_CELL_CELL_ADHESION | -0.22233 | -0.01257 | -3.96193 | 0.00023 | 0.023312 | 0.496734 |
| GO_NEGATIVE_REGULATION_OF_ERAD_PATHWAY | -0.15594 | -0.00504 | -3.95694 | 0.000233 | 0.023312 | 0.482318 |
| GO_ANTIGEN_PROCESSING_AND_PRESENTATION | 0.12422 | 0.007184 | 3.950441 | 0.000238 | 0.023412 | 0.463552 |
| GO_OLIGOSACCHARYLTRANSFERASE_COMPLEX | 0.290495 | 0.051039 | 3.945277 | 0.000242 | 0.023419 | 0.448654 |
| GO_REGULATION_OF_SHORT_TERM_NEURONAL_SYNAPTIC_PLASTICITY | -0.20545 | -0.00959 | -3.93202 | 0.000253 | 0.024045 | 0.410445 |
| GO_NEGATIVE_REGULATION_OF_RYANODINE_SENSITIVE_CALCIUM_RELEASE_CHANNEL_ACTIVITY | 0.216158 | 0.005728 | 3.900619 | 0.000279 | 0.026026 | 0.320227 |
| GO_REGULATION_OF_ACTION_POTENTIAL | -0.11974 | 0.002538 | -3.89041 | 0.000289 | 0.026026 | 0.29098 |
| GO_CHROMATIN_SILENCING_AT_RDNA | 0.195189 | -0.00387 | 3.88798 | 0.000291 | 0.026026 | 0.284015 |
| GO_NEURAL_NUCLEUS_DEVELOPMENT | 0.110257 | 0.004407 | 3.887856 | 0.000291 | 0.026026 | 0.283662 |
| GO_DEOXYRIBONUCLEOTIDE_METABOLIC_PROCESS | 0.155503 | 0.002965 | 3.876701 | 0.000301 | 0.026568 | 0.251754 |
| GO_MAINTENANCE_OF_PROTEIN_LOCALIZATION_IN_ENDOPLASMIC_RETICULUM | -0.19818 | -0.00666 | -3.86071 | 0.000317 | 0.027215 | 0.206096 |
| GO_REGULATION_OF_ER_TO_GOLGI_VESICLE_MEDIATED_TRANSPORT | 0.159011 | 0.004148 | 3.859973 | 0.000318 | 0.027215 | 0.203993 |
| GO_MICROFILAMENT_MOTOR_ACTIVITY | -0.18691 | -0.01381 | -3.85331 | 0.000325 | 0.027404 | 0.185005 |
| GO_TUMOR_NECROSIS_FACTOR_MEDIATED_SIGNALING_PATHWAY | 0.116578 | 0.009548 | 3.828473 | 0.000351 | 0.028831 | 0.11435 |
| GO_PHOSPHATIDYLSERINE_METABOLIC_PROCESS | -0.16105 | -0.01111 | -3.80983 | 0.000372 | 0.030037 | 0.061484 |
| GO_PROTEASOME_REGULATORY_PARTICLE_BASE_SUBCOMPLEX | 0.260739 | 0.04134 | 3.806897 | 0.000376 | 0.030037 | 0.053173 |
| GO_PEPTIDASE_COMPLEX | 0.162407 | 0.025109 | 3.801749 | 0.000382 | 0.030126 | 0.038603 |
| GO_REGULATION_OF_AMYLOID_BETA_FORMATION | -0.14322 | 0.006266 | -3.77322 | 0.000418 | 0.032523 | -0.04195 |
| GO_GUANYL_NUCLEOTIDE_EXCHANGE_FACTOR_COMPLEX | 0.174896 | 0.015951 | 3.763355 | 0.000431 | 0.033114 | -0.06972 |
| GO_ENDOPLASMIC_RETICULUM_QUALITY_CONTROL_COMPARTMENT | -0.18086 | -0.0203 | -3.74567 | 0.000455 | 0.034555 | -0.11943 |
| GO_AXO_DENDRITIC_TRANSPORT | 0.111471 | 0.006972 | 3.741585 | 0.000461 | 0.034561 | -0.13088 |
| GO_PROTEIN_DISULFIDE_OXIDOREDUCTASE_ACTIVITY | 0.150386 | 0.009477 | 3.723546 | 0.000488 | 0.036003 | -0.18141 |
| GO_SNORNA_BINDING | 0.218438 | 0.018732 | 3.720572 | 0.000492 | 0.036003 | -0.18973 |
| GO_CYTIDINE_DEAMINASE_ACTIVITY | 0.216336 | 0.016701 | 3.711741 | 0.000506 | 0.036561 | -0.21441 |
| GO_POSITIVE_REGULATION_OF_FATTY_ACID_TRANSPORT | -0.18562 | -0.00217 | -3.70761 | 0.000513 | 0.036593 | -0.22595 |
| GO_DNA_LIGATION | 0.18931 | -0.00363 | 3.7034 | 0.000519 | 0.036639 | -0.23769 |
| GO_REGULATION_OF_DNA_DAMAGE_CHECKPOINT | 0.162601 | 0.00686 | 3.693446 | 0.000536 | 0.03735 | -0.26543 |
| GO_REGULATION_OF_TRANSCRIPTION_INVOLVED_IN_CELL_FATE_COMMITMENT | -0.18935 | -0.03194 | -3.68769 | 0.000545 | 0.037586 | -0.28144 |
| GO_TUMOR_NECROSIS_FACTOR_SECRETION | 0.141794 | 0.024264 | 3.672224 | 0.000572 | 0.038984 | -0.32444 |
| GO_ELECTRON_TRANSFER_ACTIVITY | 0.118258 | 0.012191 | 3.662657 | 0.000589 | 0.03943 | -0.35098 |
| GO_TRANSLATION_REPRESSOR_ACTIVITY | 0.145712 | 0.012346 | 3.657038 | 0.0006 | 0.03943 | -0.36655 |
| GO_ANTIGEN_PROCESSING_AND_PRESENTATION_OF_PEPTIDE_ANTIGEN | 0.112536 | 0.011006 | 3.656925 | 0.0006 | 0.03943 | -0.36686 |
| GO_REGULATION_OF_ICOSANOID_SECRETION | -0.19166 | 0.003698 | -3.65417 | 0.000605 | 0.03943 | -0.3745 |
| GO_PHOSPHATIDYLINOSITOL_KINASE_ACTIVITY | -0.16809 | -0.01616 | -3.64727 | 0.000618 | 0.039769 | -0.39359 |
| GO_ERBB2_SIGNALING_PATHWAY | -0.15246 | 0.014837 | -3.64397 | 0.000624 | 0.039769 | -0.40271 |
| GO_NUCLEOTIDE_BINDING_DOMAIN_LEUCINE_RICH_REPEAT_CONTAINING_RECEPTOR_SIGNALING_PATHWAY | -0.12783 | 0.003304 | -3.63865 | 0.000635 | 0.039769 | -0.41741 |
| GO_NEGATIVE_REGULATION_OF_PROTEIN_DEPHOSPHORYLATION | -0.11756 | 0.002827 | -3.6376 | 0.000637 | 0.039769 | -0.42031 |
| GO_CERAMIDE_CATABOLIC_PROCESS | 0.164951 | 0.014133 | 3.605249 | 0.000703 | 0.043228 | -0.50942 |
| GO_BICARBONATE_TRANSPORT | -0.14636 | -0.00262 | -3.60378 | 0.000707 | 0.043228 | -0.51346 |
| GO_PHASIC_SMOOTH_MUSCLE_CONTRACTION | 0.131617 | 0.016236 | 3.592093 | 0.000732 | 0.044352 | -0.54552 |
| GO_DYNEIN_HEAVY_CHAIN_BINDING | -0.20465 | 0.004688 | -3.57889 | 0.000763 | 0.045719 | -0.58167 |
| GO_TRANSCRIPTION_COACTIVATOR_BINDING | -0.14172 | -0.0199 | -3.56558 | 0.000794 | 0.046244 | -0.61807 |
| GO_CELL_MIGRATION_IN_HINDBRAIN | -0.20099 | -0.02159 | -3.56148 | 0.000804 | 0.046362 | -0.62925 |
| GO_PHOSPHATIDYLINOSITOL_4_5_BISPHOSPHATE_PHOSPHATASE_ACTIVITY | -0.16652 | -0.01989 | -3.55812 | 0.000813 | 0.046393 | -0.63841 |
| GO_PROTON_CHANNEL_ACTIVITY | 0.172343 | 0.022552 | 3.55439 | 0.000822 | 0.046481 | -0.64858 |
| GO_ARGININE_METABOLIC_PROCESS | 0.156646 | 0.018125 | 3.547355 | 0.00084 | 0.046892 | -0.66774 |
| GO_COBALAMIN_METABOLIC_PROCESS | -0.14878 | -0.00382 | -3.54536 | 0.000845 | 0.046892 | -0.67316 |
| GO_EMBRYONIC_HINDLIMB_MORPHOGENESIS | -0.13537 | -0.00634 | -3.52326 | 0.000903 | 0.04969 | -0.73318 |
| GO_PROTEASOME_ACCESSORY_COMPLEX | 0.235317 | 0.044656 | 3.520037 | 0.000912 | 0.049722 | -0.74193 |
| GO_FATTY_ACID_TRANSPORT | -0.1034 | -0.00184 | -3.51329 | 0.000931 | 0.050294 | -0.76021 |
| GO_GLYCOLIPID_BINDING | 0.138639 | -0.00799 | 3.50945 | 0.000942 | 0.050427 | -0.77059 |
| GO_CULLIN_FAMILY_PROTEIN_BINDING | 0.143344 | -0.00086 | 3.502557 | 0.000962 | 0.050651 | -0.78922 |
| GO_MITOGEN_ACTIVATED_PROTEIN_KINASE_BINDING | -0.13654 | -0.00475 | -3.47814 | 0.001036 | 0.052566 | -0.85504 |
| GO_GLYCOSYL_COMPOUND_CATABOLIC_PROCESS | 0.115203 | 0.009317 | 3.477711 | 0.001037 | 0.052566 | -0.8562 |
| GO_PROTEIN_TRANSMEMBRANE_TRANSPORT | 0.102854 | 0.012518 | 3.472529 | 0.001053 | 0.052566 | -0.87014 |
| GO_REGULATION_OF_TRANSCRIPTION_INITIATION_FROM_RNA_POLYMERASE_II_PROMOTER | 0.156851 | 0.009903 | 3.470811 | 0.001059 | 0.052566 | -0.87476 |
| GO_REGULATION_OF_CARDIAC_MUSCLE_CONTRACTION_BY_REGULATION_OF_THE_RELEASE_OF_SEQUESTERED_CALCIUM_ION | 0.156291 | -0.00934 | 3.469354 | 0.001063 | 0.052566 | -0.87867 |
| GO_DE_NOVO_PROTEIN_FOLDING | 0.137619 | 0.006938 | 3.467678 | 0.001069 | 0.052566 | -0.88317 |
| GO_REGULATION_OF_VENTRICULAR_CARDIAC_MUSCLE_CELL_ACTION_POTENTIAL | -0.18105 | 0.000348 | -3.46556 | 0.001076 | 0.052566 | -0.88887 |
| GO_FATTY_ACID_DERIVATIVE_TRANSPORT | -0.1207 | -0.01074 | -3.46466 | 0.001078 | 0.052566 | -0.89128 |
| GO_NEGATIVE_REGULATION_OF_CALCIUM_ION_TRANSPORT_INTO_CYTOSOL | 0.171949 | 0.024087 | 3.453615 | 0.001115 | 0.053609 | -0.92089 |
| GO_OXIDOREDUCTASE_ACTIVITY_ACTING_ON_A_SULFUR_GROUP_OF_DONORS | 0.127668 | 0.017899 | 3.452761 | 0.001118 | 0.053609 | -0.92318 |
| GO_STRESS_GRANULE_ASSEMBLY | -0.18217 | -0.00601 | -3.44617 | 0.00114 | 0.054063 | -0.94084 |
| GO_PHOSPHATIDYLETHANOLAMINE_ACYL_CHAIN_REMODELING | -0.12161 | 0.004436 | -3.4414 | 0.001157 | 0.054063 | -0.9536 |
| GO_ANTIGEN_PROCESSING_AND_PRESENTATION_OF_EXOGENOUS_PEPTIDE_ANTIGEN_VIA_MHC_CLASS_I | 0.162854 | 0.015891 | 3.437402 | 0.00117 | 0.054063 | -0.96427 |
| GO_INOSITOL_TRISPHOSPHATE_METABOLIC_PROCESS | -0.11346 | -0.00448 | -3.43688 | 0.001172 | 0.054063 | -0.96567 |
| GO_REGULATION_OF_CARDIAC_MUSCLE_CELL_ACTION_POTENTIAL | -0.14438 | -0.00545 | -3.43362 | 0.001184 | 0.054176 | -0.97436 |
| GO_VASCULAR_SMOOTH_MUSCLE_CELL_DIFFERENTIATION | -0.11892 | -0.00832 | -3.42326 | 0.001221 | 0.055459 | -1.002 |
| GO_THREONINE_TYPE_PEPTIDASE_ACTIVITY | 0.185911 | 0.022436 | 3.416325 | 0.001247 | 0.055859 | -1.02047 |
| GO_PYRIMIDINE_RIBONUCLEOSIDE_METABOLIC_PROCESS | 0.183984 | 0.009815 | 3.415826 | 0.001249 | 0.055859 | -1.0218 |
| GO_HEMATOPOIETIC_STEM_CELL_DIFFERENTIATION | 0.119395 | 0.008042 | 3.409864 | 0.001271 | 0.056442 | -1.03766 |
| GO_TRANSCRIPTION_COFACTOR_BINDING | -0.12709 | -0.00842 | -3.39411 | 0.001332 | 0.058647 | -1.07948 |
| GO_ENDOPEPTIDASE_COMPLEX | 0.164113 | 0.024333 | 3.392073 | 0.00134 | 0.058647 | -1.08488 |
| GO_PHAGOCYTOSIS_RECOGNITION | 0.151017 | 0.011019 | 3.389027 | 0.001352 | 0.058753 | -1.09295 |
| GO_DEOXYRIBONUCLEOTIDE_BIOSYNTHETIC_PROCESS | 0.218438 | -0.01012 | 3.383461 | 0.001375 | 0.059304 | -1.10768 |
| GO_NEGATIVE_REGULATION_OF_NEURAL_PRECURSOR_CELL_PROLIFERATION | -0.16894 | -0.00431 | -3.3758 | 0.001407 | 0.059987 | -1.12795 |
| GO_WNT_SIGNALING_PATHWAY_CALCIUM_MODULATING_PATHWAY | -0.10508 | -0.00402 | -3.36901 | 0.001435 | 0.060419 | -1.14587 |
| GO_POSITIVE_REGULATION_OF_MITOCHONDRIAL_TRANSLATION | 0.19145 | 0.015417 | 3.361089 | 0.001469 | 0.060419 | -1.16676 |
| GO_POSITIVE_REGULATION_OF_PHOSPHATASE_ACTIVITY | 0.139379 | 0.00468 | 3.360953 | 0.00147 | 0.060419 | -1.16712 |
| GO_SPINDLE_MIDZONE | 0.162745 | 0.004259 | 3.360896 | 0.00147 | 0.060419 | -1.16727 |
| GO_AMYLOID_BETA_FORMATION | -0.1449 | 0.012464 | -3.36063 | 0.001471 | 0.060419 | -1.16796 |
| GO_INSULIN_RECEPTOR_BINDING | -0.14742 | 0.005187 | -3.3412 | 0.001559 | 0.06313 | -1.21909 |
| GO_REGULATION_OF_B_CELL_APOPTOTIC_PROCESS | 0.192295 | -0.00497 | 3.336934 | 0.001578 | 0.0635 | -1.23027 |
| GO_PROTON_TRANSPORTING_ATP_SYNTHASE_ACTIVITY_ROTATIONAL_MECHANISM | 0.191636 | 0.023393 | 3.332736 | 0.001598 | 0.063864 | -1.24128 |
| GO_PROTEASOME_CORE_COMPLEX | 0.196688 | 0.021797 | 3.323966 | 0.00164 | 0.065021 | -1.26425 |
| GO_PEPTIDYL_ARGININE_MODIFICATION | 0.12642 | 0.003126 | 3.322149 | 0.001649 | 0.065021 | -1.269 |
| GO_NEGATIVE_REGULATION_OF_PHAGOCYTOSIS | 0.19218 | 0.019588 | 3.317144 | 0.001673 | 0.065169 | -1.28209 |
| GO_DEAMINASE_ACTIVITY | 0.125053 | 0.008711 | 3.314736 | 0.001685 | 0.065169 | -1.28838 |
| GO_NEGATIVE_REGULATION_OF_CELL_CYCLE_G2_M_PHASE_TRANSITION | 0.131068 | 0.009715 | 3.31219 | 0.001698 | 0.065238 | -1.29503 |
| GO_INTRINSIC_APOPTOTIC_SIGNALING_PATHWAY_IN_RESPONSE_TO_DNA_DAMAGE_BY_P53_CLASS_MEDIATOR | 0.14142 | 0.01288 | 3.302819 | 0.001745 | 0.066211 | -1.31948 |
| GO_DYNEIN_LIGHT_INTERMEDIATE_CHAIN_BINDING | -0.15005 | -0.01366 | -3.29546 | 0.001783 | 0.067045 | -1.33864 |
| GO_GLIAL_CELL_FATE_COMMITMENT | -0.13994 | -0.01509 | -3.29329 | 0.001794 | 0.067045 | -1.34429 |
| GO_BASAL_PLASMA_MEMBRANE | -0.12349 | -0.00986 | -3.29214 | 0.001801 | 0.067045 | -1.34728 |
| GO_LAMELLAR_BODY | -0.15356 | -0.01052 | -3.26418 | 0.001954 | 0.071886 | -1.41981 |
| GO_FATTY_ACID_BINDING | 0.104526 | 0.006278 | 3.261576 | 0.001969 | 0.071886 | -1.42656 |
| GO_REGULATION_OF_CHOLESTEROL_EFFLUX | -0.14781 | 0.008907 | -3.2599 | 0.001979 | 0.071886 | -1.43089 |
| GO_RESPONSE_TO_IMMOBILIZATION_STRESS | -0.13464 | -0.00221 | -3.24268 | 0.00208 | 0.074246 | -1.47533 |
| GO_MYELOID_CELL_APOPTOTIC_PROCESS | 0.143872 | 0.021361 | 3.242626 | 0.002081 | 0.074246 | -1.47547 |
| GO_U5_SNRNP | 0.194911 | 0.015115 | 3.238718 | 0.002104 | 0.07465 | -1.48554 |
| GO_BICARBONATE_TRANSMEMBRANE_TRANSPORTER_ACTIVITY | -0.15502 | 0.013102 | -3.23641 | 0.002119 | 0.074709 | -1.49147 |
| KEGG_PROTEASOME | 0.1849 | 0.019933 | 3.486898 | 0.000986 | 0.077651 | -0.77442 |
| KEGG_PROTEIN_EXPORT | 0.191535 | 0.022806 | 3.398679 | 0.001287 | 0.077651 | -1.00603 |
| GO_TRANSLATIONAL_ELONGATION | 0.123071 | 0.015251 | 3.206681 | 0.002309 | 0.080485 | -1.56775 |
| GO_LIGASE_ACTIVITY_FORMING_CARBON_NITROGEN_BONDS | 0.124245 | 0.009166 | 3.197876 | 0.002369 | 0.082013 | -1.59025 |
| GO_CHEMOREPELLENT_ACTIVITY | 0.158026 | -0.01778 | 3.196169 | 0.00238 | 0.082013 | -1.59461 |
| GO_REGULATION_OF_COFACTOR_METABOLIC_PROCESS | 0.113354 | 0.025807 | 3.192689 | 0.002404 | 0.082367 | -1.60349 |
| GO_PHOSPHATIDYLINOSITOL_3_KINASE_ACTIVITY | -0.14016 | -0.00798 | -3.18215 | 0.002479 | 0.084426 | -1.63035 |
| GO_SEQUENCE_SPECIFIC_SINGLE_STRANDED_DNA_BINDING | 0.13119 | 0.016567 | 3.176606 | 0.002518 | 0.085298 | -1.64444 |
| GO_VASCULAR_ASSOCIATED_SMOOTH_MUSCLE_CELL_MIGRATION | -0.17264 | -0.00804 | -3.17085 | 0.00256 | 0.085751 | -1.65906 |
| GO_PROTEIN_DEMANNOSYLATION | -0.15409 | -0.02079 | -3.16752 | 0.002585 | 0.086097 | -1.66753 |
| GO_POSITIVE_REGULATION_OF_SMAD_PROTEIN_SIGNAL_TRANSDUCTION | 0.197974 | 0.013129 | 3.162831 | 0.00262 | 0.086097 | -1.67941 |
| GO_DEFINITIVE_HEMOPOIESIS | -0.14192 | -0.00992 | -3.16153 | 0.00263 | 0.086097 | -1.68272 |
| GO_PEPTIDE_TRANSMEMBRANE_TRANSPORTER_ACTIVITY | 0.111957 | 0.018217 | 3.152336 | 0.0027 | 0.087498 | -1.70599 |
| GO_STEREOCILIUM_TIP | -0.15757 | -0.00949 | -3.14554 | 0.002753 | 0.088512 | -1.72317 |
| GO_MITOCHONDRION_MORPHOGENESIS | 0.144251 | 0.014414 | 3.143896 | 0.002766 | 0.088512 | -1.72732 |
| GO_REGULATION_OF_HEMATOPOIETIC_PROGENITOR_CELL_DIFFERENTIATION | 0.113047 | 0.012074 | 3.140592 | 0.002792 | 0.088512 | -1.73566 |
| GO_PROTEASOMAL_UBIQUITIN_INDEPENDENT_PROTEIN_CATABOLIC_PROCESS | 0.170877 | 0.013119 | 3.136397 | 0.002826 | 0.088512 | -1.74625 |
| GO_DETERMINATION_OF_ADULT_LIFESPAN | -0.13474 | -0.01225 | -3.13613 | 0.002828 | 0.088512 | -1.74691 |
| GO_NEGATIVE_REGULATION_OF_RESPONSE_TO_DRUG | 0.102399 | 0.011268 | 3.128049 | 0.002894 | 0.088512 | -1.76727 |
| GO_POSITIVE_REGULATION_OF_PHOSPHOPROTEIN_PHOSPHATASE_ACTIVITY | 0.154591 | -0.00038 | 3.127822 | 0.002896 | 0.088512 | -1.76784 |
| GO_CAJAL_BODY | 0.145816 | 0.004613 | 3.126695 | 0.002905 | 0.088512 | -1.77068 |
| GO_AMYLOID_PRECURSOR_PROTEIN_BIOSYNTHETIC_PROCESS | -0.14055 | -0.00536 | -3.12495 | 0.00292 | 0.088512 | -1.77508 |
| GO_HYALURONIC_ACID_BINDING | 0.168952 | 0.001485 | 3.119419 | 0.002966 | 0.088512 | -1.78897 |
| GO_POSITIVE_REGULATION_OF_MYELOID_LEUKOCYTE_CYTOKINE_PRODUCTION_INVOLVED_IN_IMMUNE_RESPONSE | 0.164523 | 0.001462 | 3.11897 | 0.00297 | 0.088512 | -1.7901 |
| GO_NUCLEAR_CYCLIN_DEPENDENT_PROTEIN_KINASE_HOLOENZYME_COMPLEX | -0.1738 | -0.00938 | -3.11743 | 0.002983 | 0.088512 | -1.79396 |
| GO_MITOCHONDRIAL_TRANSLATIONAL_TERMINATION | 0.143297 | 0.018007 | 3.117331 | 0.002984 | 0.088512 | -1.79422 |
| GO_POSITIVE_REGULATION_OF_CAMP_MEDIATED_SIGNALING | -0.14012 | -0.01176 | -3.11681 | 0.002988 | 0.088512 | -1.79552 |
| GO_QUINONE_METABOLIC_PROCESS | 0.130465 | 0.015102 | 3.114645 | 0.003007 | 0.088512 | -1.80096 |
| KEGG_HYPERTROPHIC_CARDIOMYOPATHY_HCM | -0.10131 | -0.00299 | -3.25708 | 0.001959 | 0.088646 | -1.37007 |
| GO_POSITIVE_REGULATION_OF_CALCIUM_ION_DEPENDENT_EXOCYTOSIS | -0.14633 | -0.02641 | -3.11026 | 0.003045 | 0.088737 | -1.81196 |
| GO_SKELETAL_MUSCLE_ADAPTATION | -0.13465 | 0.001559 | -3.10974 | 0.003049 | 0.088737 | -1.81327 |
| GO_POSITIVE_REGULATION_OF_PROTEIN_AUTOPHOSPHORYLATION | 0.108802 | 0.011022 | 3.10757 | 0.003068 | 0.088854 | -1.8187 |
| GO_REGULATION_OF_INTRACELLULAR_ESTROGEN_RECEPTOR_SIGNALING_PATHWAY | -0.11125 | -0.00181 | -3.1037 | 0.003102 | 0.089125 | -1.82839 |
| GO_POSITIVE_REGULATION_OF_CELLULAR_RESPONSE_TO_INSULIN_STIMULUS | -0.11431 | -0.0145 | -3.0979 | 0.003153 | 0.089377 | -1.8429 |
| GO_DIVALENT_INORGANIC_ANION_HOMEOSTASIS | -0.15921 | -0.0081 | -3.0971 | 0.003161 | 0.089377 | -1.84491 |
| GO_ARACHIDONIC_ACID_SECRETION | -0.11204 | -0.00946 | -3.09272 | 0.0032 | 0.089809 | -1.85586 |
| GO_CHEMOSENSORY_BEHAVIOR | -0.13351 | 0.006441 | -3.09156 | 0.003211 | 0.089809 | -1.85874 |
| GO_SEX_CHROMOSOME | 0.15974 | 0.001302 | 3.089495 | 0.00323 | 0.089809 | -1.86391 |
| GO_SOLUTE_ANION_ANTIPORTER_ACTIVITY | -0.11183 | -0.00375 | -3.07794 | 0.003337 | 0.091276 | -1.89272 |
| GO_OLIGOSACCHARIDE_BINDING | 0.190453 | 0.004525 | 3.076598 | 0.00335 | 0.091276 | -1.89605 |
| GO_NEUTROPHIL_HOMEOSTASIS | 0.172696 | 0.025598 | 3.071807 | 0.003395 | 0.092102 | -1.90797 |
| GO_PHOSPHATIDYLSERINE_ACYL_CHAIN_REMODELING | -0.14427 | -0.00427 | -3.0631 | 0.00348 | 0.093573 | -1.9296 |
| GO_POSITIVE_REGULATION_OF_EPIDERMAL_CELL_DIFFERENTIATION | -0.11532 | 0.006814 | -3.06148 | 0.003496 | 0.093573 | -1.93361 |
| GO_NEGATIVE_REGULATION_OF_CELL_MIGRATION_INVOLVED_IN_SPROUTING_ANGIOGENESIS | 0.149774 | -0.00512 | 3.061414 | 0.003496 | 0.093573 | -1.93378 |
| GO_TRANSCRIPTION_INITIATION_FROM_RNA_POLYMERASE_I_PROMOTER | 0.104828 | -0.00172 | 3.058952 | 0.003521 | 0.093806 | -1.93989 |
| GO_AMMONIUM_TRANSMEMBRANE_TRANSPORT | 0.137627 | 0.020659 | 3.0443 | 0.003669 | 0.097248 | -1.97616 |
| GO_NEGATIVE_REGULATION_OF_POTASSIUM_ION_TRANSPORT | 0.114098 | 0.024281 | 3.041438 | 0.003698 | 0.097248 | -1.98323 |
| GO_RETINOIC_ACID_RECEPTOR_BINDING | -0.10142 | -0.00263 | -3.03183 | 0.0038 | 0.099038 | -2.00694 |
| GO_REGULATION_OF_VIRAL_INDUCED_CYTOPLASMIC_PATTERN_RECOGNITION_RECEPTOR_SIGNALING_PATHWAY | -0.14033 | 0.019619 | -3.02569 | 0.003866 | 0.099904 | -2.02206 |
| GO_MYOBLAST_MIGRATION | -0.14543 | 0.011979 | -3.02563 | 0.003866 | 0.099904 | -2.0222 |
| GO_NEGATIVE_REGULATION_OF_TRANSLATIONAL_INITIATION | 0.11624 | 0.029651 | 3.021703 | 0.003909 | 0.100576 | -2.03187 |
| GO_PRE_MIRNA_PROCESSING | -0.15256 | -0.02195 | -3.01995 | 0.003928 | 0.100638 | -2.03618 |
| GO_PROTEIN_REFOLDING | 0.13392 | -0.00403 | 3.015479 | 0.003978 | 0.101471 | -2.04716 |
| GO_REGULATION_OF_LYSOSOMAL_LUMEN_PH | 0.137334 | 0.004726 | 3.008935 | 0.004051 | 0.102795 | -2.06322 |
| GO_POSITIVE_REGULATION_OF_ANTIGEN_RECEPTOR_MEDIATED_SIGNALING_PATHWAY | 0.165214 | 0.019033 | 3.007806 | 0.004064 | 0.102795 | -2.06599 |
| GO_PEPTIDYL_METHIONINE_MODIFICATION | 0.137234 | 0.016067 | 2.999928 | 0.004154 | 0.104638 | -2.08528 |
| GO_INTRINSIC_COMPONENT_OF_NUCLEAR_INNER_MEMBRANE | -0.13273 | -0.00057 | -2.99311 | 0.004234 | 0.105755 | -2.10194 |
| GO_LATERAL_PLASMA_MEMBRANE | -0.10499 | 0.005134 | -2.97446 | 0.004459 | 0.11092 | -2.14741 |
| GO_DNA_DEMETHYLATION | 0.113382 | 0.001157 | 2.970878 | 0.004504 | 0.111566 | -2.15613 |
| GO_NEGATIVE_REGULATION_OF_SIGNAL_TRANSDUCTION_IN_ABSENCE_OF_LIGAND | 0.102556 | -0.00137 | 2.952996 | 0.004732 | 0.115793 | -2.1995 |
| GO_MAMMARY_GLAND_EPITHELIAL_CELL_PROLIFERATION | -0.10258 | -0.00195 | -2.95141 | 0.004753 | 0.115829 | -2.20334 |
| GO_GASTRIC_ACID_SECRETION | -0.15351 | 0.001264 | -2.94959 | 0.004777 | 0.115942 | -2.20774 |
| GO_POSITIVE_REGULATION_OF_NEUROINFLAMMATORY_RESPONSE | 0.201324 | -0.00504 | 2.946634 | 0.004816 | 0.116149 | -2.21488 |
| GO_SNRNA_PROCESSING | 0.130374 | 0.00979 | 2.946022 | 0.004824 | 0.116149 | -2.21637 |
| GO_TRANSLATIONAL_TERMINATION | 0.126503 | 0.016607 | 2.943955 | 0.004852 | 0.116345 | -2.22136 |
| GO_REGULATION_OF_TRANSCRIPTION_FROM_RNA_POLYMERASE_II_PROMOTER_IN_RESPONSE_TO_HYPOXIA | 0.112701 | 0.009596 | 2.938681 | 0.004923 | 0.11758 | -2.23409 |
| GO_REGULATION_OF_GTP_BINDING | 0.151647 | -0.0036 | 2.933043 | 0.005 | 0.118946 | -2.24768 |
| GO_INTRINSIC_COMPONENT_OF_MITOCHONDRIAL_OUTER_MEMBRANE | -0.13053 | 0.000476 | -2.93043 | 0.005036 | 0.119333 | -2.25398 |
| GO_RESPONSE_TO_ATP | -0.10342 | 0.004314 | -2.92689 | 0.005085 | 0.120024 | -2.26249 |
| GO_REGULATION_OF_DNA_TEMPLATED_TRANSCRIPTION_INITIATION | 0.117598 | 0.004651 | 2.924166 | 0.005124 | 0.120148 | -2.26904 |
| GO_NEGATIVE_REGULATION_OF_LIPASE_ACTIVITY | -0.14608 | 7.42E-05 | -2.92313 | 0.005138 | 0.120148 | -2.27154 |
| GO_MODIFIED_AMINO_ACID_TRANSMEMBRANE_TRANSPORTER_ACTIVITY | 0.121374 | 0.009056 | 2.919508 | 0.005189 | 0.120585 | -2.28023 |
| GO_REGULATION_OF_INTEGRIN_MEDIATED_SIGNALING_PATHWAY | 0.135859 | 0.019378 | 2.914665 | 0.005259 | 0.121727 | -2.29186 |
| GO_DETECTION_OF_OTHER_ORGANISM | -0.159 | -0.01261 | -2.9087 | 0.005346 | 0.123259 | -2.30616 |
| GO_POSITIVE_REGULATION_OF_RELEASE_OF_SEQUESTERED_CALCIUM_ION_INTO_CYTOSOL | 0.130052 | 0.015295 | 2.904646 | 0.005405 | 0.124113 | -2.31586 |
| GO_AXONEMAL_DYNEIN_COMPLEX | -0.21819 | -0.04428 | -2.90338 | 0.005424 | 0.124113 | -2.31889 |
| GO_SNRNA_3_END_PROCESSING | 0.154295 | 0.007638 | 2.894133 | 0.005563 | 0.125645 | -2.34098 |
| GO_PEPTIDE_DISULFIDE_OXIDOREDUCTASE_ACTIVITY | 0.171599 | 0.010083 | 2.893782 | 0.005568 | 0.125645 | -2.34182 |
| GO_REGULATION_OF_VASCULAR_SMOOTH_MUSCLE_CELL_DIFFERENTIATION | -0.12148 | -0.00685 | -2.89318 | 0.005578 | 0.125645 | -2.34327 |
| GO_NEGATIVE_REGULATION_OF_PROTEIN_LOCALIZATION_TO_NUCLEUS | 0.112425 | 0.002081 | 2.891858 | 0.005598 | 0.125645 | -2.34641 |
| GO_POSITIVE_REGULATION_OF_SODIUM_ION_TRANSMEMBRANE_TRANSPORT | -0.15658 | -0.0024 | -2.89103 | 0.00561 | 0.125645 | -2.34839 |
| GO_LIVER_MORPHOGENESIS | -0.11859 | -0.0125 | -2.88898 | 0.005642 | 0.125645 | -2.35326 |
| GO_AMYLOID_BETA_METABOLIC_PROCESS | -0.11698 | 0.010449 | -2.88866 | 0.005647 | 0.125645 | -2.35404 |
| GO_NEGATIVE_REGULATION_OF_DNA_REPLICATION | 0.116042 | 0.007668 | 2.885041 | 0.005703 | 0.125645 | -2.36266 |
| GO_MITOCHONDRIAL_FISSION | 0.142423 | 0.002447 | 2.882681 | 0.00574 | 0.125645 | -2.36828 |
| GO_REGULATION_OF_INTRINSIC_APOPTOTIC_SIGNALING_PATHWAY_BY_P53_CLASS_MEDIATOR | 0.122508 | 0.002635 | 2.882497 | 0.005743 | 0.125645 | -2.36871 |
| GO_CELLULAR_CARBOHYDRATE_CATABOLIC_PROCESS | -0.1261 | -0.00294 | -2.87607 | 0.005844 | 0.126649 | -2.38399 |
| GO_POSITIVE_REGULATION_OF_SEQUESTERING_OF_CALCIUM_ION | 0.162863 | 0.016938 | 2.875581 | 0.005852 | 0.126649 | -2.38516 |
| GO_NEGATIVE_REGULATION_OF_OLIGODENDROCYTE_DIFFERENTIATION | -0.13702 | -0.0006 | -2.87051 | 0.005933 | 0.127031 | -2.39719 |
| GO_MELANIN_METABOLIC_PROCESS | -0.11767 | -0.00678 | -2.85997 | 0.006106 | 0.130258 | -2.42218 |
| GO_2_IRON_2_SULFUR_CLUSTER_BINDING | 0.124222 | 0.005547 | 2.849388 | 0.006283 | 0.132678 | -2.44719 |
| GO_GLYCOSIDE_METABOLIC_PROCESS | 0.125902 | -0.00598 | 2.845037 | 0.006358 | 0.132678 | -2.45745 |
| GO_REGULATION_OF_POSTSYNAPTIC_MEMBRANE_ORGANIZATION | -0.11467 | 0.010189 | -2.84448 | 0.006367 | 0.132678 | -2.45877 |
| GO_CELL_DIFFERENTIATION_INVOLVED_IN_METANEPHROS_DEVELOPMENT | -0.10973 | -0.00334 | -2.84402 | 0.006375 | 0.132678 | -2.45985 |
| GO_REGULATION_OF_NEUTROPHIL_CHEMOTAXIS | 0.129567 | -0.00237 | 2.841979 | 0.00641 | 0.132678 | -2.46466 |
| GO_SECONDARY_METABOLITE_BIOSYNTHETIC_PROCESS | -0.10464 | 0.004615 | -2.84027 | 0.00644 | 0.132678 | -2.46869 |
| GO_REGULATION_OF_NUCLEASE_ACTIVITY | 0.136841 | 0.02378 | 2.831856 | 0.006588 | 0.134895 | -2.48848 |
| GO_REGULATION_OF_TRANSCRIPTION_FROM_RNA_POLYMERASE_II_PROMOTER_IN_RESPONSE_TO_OXIDATIVE_STRESS | 0.129721 | 0.002423 | 2.831461 | 0.006595 | 0.134895 | -2.48941 |
| GO_REGULATION_OF_EXTRINSIC_APOPTOTIC_SIGNALING_PATHWAY_IN_ABSENCE_OF_LIGAND | 0.101192 | 0.000201 | 2.830334 | 0.006615 | 0.134895 | -2.49206 |
| GO_LARGE_RIBOSOMAL_SUBUNIT | 0.136285 | 0.009134 | 2.828385 | 0.00665 | 0.135146 | -2.49663 |
| GO_RESPONSE_TO_GONADOTROPIN | 0.10938 | 0.000119 | 2.826168 | 0.00669 | 0.135315 | -2.50184 |
| GO_CLATHRIN_COAT_ASSEMBLY | -0.13484 | -0.00802 | -2.82542 | 0.006704 | 0.135315 | -2.50361 |
| GO_ACTIVATION_OF_JUN_KINASE_ACTIVITY | -0.11358 | -0.00274 | -2.8194 | 0.006813 | 0.136608 | -2.51772 |
| GO_CALCIUM_CHANNEL_INHIBITOR_ACTIVITY | 0.149405 | 0.040223 | 2.814248 | 0.006908 | 0.138051 | -2.52977 |
| GO_POTASSIUM_ION_IMPORT_ACROSS_PLASMA_MEMBRANE | -0.10534 | 0.00408 | -2.80747 | 0.007035 | 0.140123 | -2.54562 |
| GO_THYROID_HORMONE_GENERATION | -0.12433 | -0.00043 | -2.80528 | 0.007077 | 0.14046 | -2.55072 |
| GO_LAMELLIPODIUM_MEMBRANE | 0.141787 | 0.02481 | 2.803661 | 0.007108 | 0.14046 | -2.55451 |
| GO_TRANSFERASE_ACTIVITY_TRANSFERRING_ALKYL_OR_ARYL_OTHER_THAN_METHYL_GROUPS | 0.10075 | 0.015416 | 2.802777 | 0.007125 | 0.14046 | -2.55657 |
| GO_CELLULAR_RESPONSE_TO_ZINC_ION | -0.17145 | 0.000437 | -2.80098 | 0.007159 | 0.14046 | -2.56076 |
| GO_CORONARY_VASCULATURE_MORPHOGENESIS | 0.117599 | -0.00728 | 2.800422 | 0.00717 | 0.14046 | -2.56206 |
| GO_LAYER_FORMATION_IN_CEREBRAL_CORTEX | -0.1393 | -0.02249 | -2.79922 | 0.007193 | 0.14046 | -2.56487 |
| GO_HISTONE_DEUBIQUITINATION | -0.12658 | -0.00205 | -2.79705 | 0.007235 | 0.140819 | -2.56991 |
| GO_POSITIVE_REGULATION_OF_SODIUM_ION_TRANSMEMBRANE_TRANSPORTER_ACTIVITY | -0.1734 | -0.00607 | -2.79428 | 0.007289 | 0.140954 | -2.57637 |
| GO_TRANS_SYNAPTIC_SIGNALING_MODULATING_SYNAPTIC_TRANSMISSION | 0.17816 | 0.002575 | 2.792393 | 0.007326 | 0.141075 | -2.58076 |
| GO_GLUTATHIONE_TRANSFERASE_ACTIVITY | 0.141775 | -0.00299 | 2.791402 | 0.007345 | 0.141075 | -2.58306 |
| GO_CORNIFICATION | -0.11564 | -0.00173 | -2.79036 | 0.007366 | 0.141075 | -2.58549 |
| GO_LIGASE_ACTIVITY_FORMING_CARBON_SULFUR_BONDS | -0.10115 | 0.001796 | -2.78718 | 0.007429 | 0.141634 | -2.59288 |
| GO_CORONARY_VASCULATURE_DEVELOPMENT | 0.103182 | 0.002222 | 2.785317 | 0.007466 | 0.141634 | -2.59721 |
| GO_MONOVALENT_INORGANIC_ANION_HOMEOSTASIS | -0.10818 | 0.002373 | -2.78229 | 0.007526 | 0.142191 | -2.60423 |
| GO_PLATELET_DERIVED_GROWTH_FACTOR_RECEPTOR_BETA_SIGNALING_PATHWAY | -0.15154 | 0.001812 | -2.78149 | 0.007542 | 0.142191 | -2.60609 |
| GO_RNA_POLYMERASE_I_COMPLEX | 0.160665 | -0.00307 | 2.777485 | 0.007623 | 0.142821 | -2.61538 |
| GO_RNA_POLYMERASE_I_ACTIVITY | 0.160665 | -0.00307 | 2.777485 | 0.007623 | 0.142821 | -2.61538 |
| GO_MODIFICATION_OF_POSTSYNAPTIC_STRUCTURE | 0.121145 | 0.008908 | 2.773528 | 0.007704 | 0.143887 | -2.62454 |
| GO_CARBOXY_TERMINAL_DOMAIN_PROTEIN_KINASE_COMPLEX | -0.14505 | -0.01275 | -2.77137 | 0.007749 | 0.144269 | -2.62954 |
| GO_CELLULAR_RESPONSE_TO_PROSTAGLANDIN_STIMULUS | 0.103951 | 0.002913 | 2.769773 | 0.007782 | 0.144435 | -2.63323 |
| GO_FORMATION_OF_CYTOPLASMIC_TRANSLATION_INITIATION_COMPLEX | 0.158559 | 0.016763 | 2.765883 | 0.007863 | 0.145489 | -2.64223 |
| GO_PYRIMIDINE_DEOXYRIBONUCLEOTIDE_METABOLIC_PROCESS | 0.139147 | -0.004 | 2.763175 | 0.00792 | 0.145657 | -2.64848 |
| GO_RENAL_FILTRATION | 0.147041 | 0.002949 | 2.762418 | 0.007936 | 0.145657 | -2.65023 |
| GO_PHOTORECEPTOR_DISC_MEMBRANE | -0.12485 | -0.012 | -2.76199 | 0.007945 | 0.145657 | -2.65122 |
| GO_DYNEIN_COMPLEX | -0.13219 | -0.00541 | -2.75528 | 0.008088 | 0.147446 | -2.6667 |
| GO_WIDE_PORE_CHANNEL_ACTIVITY | 0.113541 | -0.00588 | 2.753414 | 0.008128 | 0.147446 | -2.67099 |
| GO_REGULATION_OF_ACTIN_FILAMENT_BASED_MOVEMENT | -0.10508 | -0.00434 | -2.75243 | 0.008149 | 0.147446 | -2.67324 |
| GO_SMN_SM_PROTEIN_COMPLEX | 0.181384 | -0.00436 | 2.751092 | 0.008178 | 0.147446 | -2.67633 |
| GO_MITOTIC_SPINDLE_ASSEMBLY | 0.101888 | 0.014367 | 2.750963 | 0.008181 | 0.147446 | -2.67663 |
| GO_3_5_CYCLIC_GMP_PHOSPHODIESTERASE_ACTIVITY | 0.124395 | 0.004111 | 2.750553 | 0.00819 | 0.147446 | -2.67757 |
| GO_AORTA_DEVELOPMENT | 0.10321 | 0.001049 | 2.746918 | 0.008269 | 0.147986 | -2.68594 |
| GO_NEGATIVE_REGULATION_OF_OXIDOREDUCTASE_ACTIVITY | 0.114306 | 0.001841 | 2.744294 | 0.008327 | 0.148438 | -2.69197 |
| GO_MACROMOLECULE_TRANSMEMBRANE_TRANSPORTER_ACTIVITY | 0.114751 | 0.01314 | 2.738055 | 0.008466 | 0.149512 | -2.70629 |
| GO_OLIGOSACCHARIDE_BIOSYNTHETIC_PROCESS | 0.117625 | -0.00583 | 2.735378 | 0.008526 | 0.149512 | -2.71243 |
| GO_MITOCHONDRIAL_LARGE_RIBOSOMAL_SUBUNIT | 0.131048 | 0.015668 | 2.733584 | 0.008567 | 0.14973 | -2.71654 |
| GO_PROTEIN_FOLDING_CHAPERONE | 0.104869 | 0.013532 | 2.728733 | 0.008677 | 0.151083 | -2.72764 |
| GO_NEGATIVE_REGULATION_OF_RELEASE_OF_CYTOCHROME_C_FROM_MITOCHONDRIA | 0.142826 | -0.01033 | 2.727983 | 0.008694 | 0.151083 | -2.72936 |
| GO_PHOSPHATIDIC_ACID_BINDING | -0.13157 | 0.001937 | -2.72567 | 0.008748 | 0.151133 | -2.73465 |
| GO_ION_TRANSMEMBRANE_TRANSPORTER_ACTIVITY_PHOSPHORYLATIVE_MECHANISM | -0.13018 | -0.00154 | -2.72274 | 0.008816 | 0.151145 | -2.74133 |
| GO_TRANSLATION_INITIATION_FACTOR_ACTIVITY | 0.115857 | 0.025534 | 2.722376 | 0.008824 | 0.151145 | -2.74217 |
| GO_ORGANELLAR_RIBOSOME | 0.130216 | 0.012051 | 2.71812 | 0.008924 | 0.151183 | -2.75189 |
| GO_MAMMARY_GLAND_EPITHELIAL_CELL_DIFFERENTIATION | -0.13273 | -0.01187 | -2.71584 | 0.008978 | 0.151183 | -2.7571 |
| GO_RNA_DECAPPING | 0.140014 | -0.00577 | 2.713577 | 0.009031 | 0.151659 | -2.76225 |
| GO_PROTEIN_KINASE_A_REGULATORY_SUBUNIT_BINDING | -0.13167 | -0.00047 | -2.709 | 0.009141 | 0.152279 | -2.77267 |
| GO_SPLEEN_DEVELOPMENT | -0.10013 | -0.00673 | -2.70852 | 0.009152 | 0.152279 | -2.77377 |
| GO_TELOMERIC_DNA_BINDING | 0.115316 | 0.011913 | 2.707795 | 0.00917 | 0.152279 | -2.77541 |
| GO_CHAPERONE_COFACTOR_DEPENDENT_PROTEIN_REFOLDING | 0.109013 | 0.002395 | 2.706385 | 0.009204 | 0.152422 | -2.77862 |
| GO_NEGATIVE_REGULATION_OF_CELLULAR_RESPONSE_TO_DRUG | 0.112579 | 0.002484 | 2.704148 | 0.009258 | 0.152899 | -2.7837 |
| GO_TRANSCRIPTION_FACTOR_TFTC_COMPLEX | 0.132576 | 0.012276 | 2.701207 | 0.00933 | 0.153243 | -2.79038 |
| GO_RRNA_BINDING | 0.142573 | 0.005449 | 2.701202 | 0.00933 | 0.153243 | -2.79039 |
| GO_RESPONSE_TO_REDOX_STATE | 0.140494 | 0.001259 | 2.698787 | 0.009389 | 0.153273 | -2.79588 |
| GO_ENDOPLASMIC_RETICULUM_MANNOSE_TRIMMING | -0.13756 | -0.02341 | -2.69833 | 0.009401 | 0.153273 | -2.79692 |
| GO_CUL3_RING_UBIQUITIN_LIGASE_COMPLEX | 0.12309 | -0.00061 | 2.694647 | 0.009492 | 0.153653 | -2.80527 |
| GO_REGULATION_OF_PROTEIN_LOCALIZATION_TO_SYNAPSE | 0.117446 | 0.01869 | 2.692946 | 0.009534 | 0.153653 | -2.80912 |
| GO_SEMAPHORIN_RECEPTOR_BINDING | 0.111071 | -0.02109 | 2.691449 | 0.009572 | 0.153843 | -2.81251 |
| GO_PHOSPHATIDYLINOSITOL_5_PHOSPHATE_BINDING | -0.1024 | -0.00856 | -2.6877 | 0.009666 | 0.154948 | -2.82101 |
| GO_ASYMMETRIC_CELL_DIVISION | -0.11728 | 0.00795 | -2.67817 | 0.00991 | 0.157592 | -2.84252 |
| GO_TRNA_THREONYLCARBAMOYLADENOSINE_METABOLIC_PROCESS | 0.164889 | -0.01079 | 2.667424 | 0.010192 | 0.161223 | -2.86675 |
| GO_CALCIUM_ION_REGULATED_EXOCYTOSIS_OF_NEUROTRANSMITTER | -0.11272 | -0.00473 | -2.66258 | 0.010322 | 0.161615 | -2.87763 |
| GO_MYD88_INDEPENDENT_TOLL_LIKE_RECEPTOR_SIGNALING_PATHWAY | -0.11129 | -0.00471 | -2.66225 | 0.010331 | 0.161615 | -2.87838 |
| GO_PROTEIN_QUALITY_CONTROL_FOR_MISFOLDED_OR_INCOMPLETELY_SYNTHESIZED_PROTEINS | -0.10863 | 0.000489 | -2.66149 | 0.010351 | 0.161615 | -2.88008 |
| GO_CELLULAR_RESPONSE_TO_PH | 0.108782 | -0.00946 | 2.657542 | 0.010458 | 0.162849 | -2.88895 |
| GO_ANGIOGENESIS_INVOLVED_IN_WOUND_HEALING | 0.128506 | 0.016358 | 2.653907 | 0.010558 | 0.16397 | -2.8971 |
| GO_GLUTAMINE_METABOLIC_PROCESS | 0.141453 | -0.00414 | 2.650505 | 0.010651 | 0.16475 | -2.90472 |
| GO_DNA_DEAMINATION | 0.150766 | 0.010602 | 2.650092 | 0.010663 | 0.16475 | -2.90565 |
| GO_LIPOPROTEIN_PARTICLE_RECEPTOR_ACTIVITY | -0.12527 | -0.00793 | -2.6474 | 0.010737 | 0.165477 | -2.91166 |
| GO_AEROBIC_RESPIRATION | 0.104202 | 0.00793 | 2.643986 | 0.010833 | 0.166461 | -2.91931 |
| GO_UBIQUINONE_METABOLIC_PROCESS | 0.143489 | 0.020193 | 2.643145 | 0.010857 | 0.166461 | -2.92119 |
| GO_PROLINE_METABOLIC_PROCESS | -0.14038 | -0.00776 | -2.63926 | 0.010967 | 0.166597 | -2.92987 |
| GO_REGULATION_OF_CELL_CYCLE_G2_M_PHASE_TRANSITION | 0.103067 | 0.00112 | 2.638903 | 0.010977 | 0.166597 | -2.93066 |
| GO_MITOCHONDRIAL_TRANSLATION | 0.119903 | 0.014749 | 2.63645 | 0.011047 | 0.166901 | -2.93613 |
| GO_DETECTION_OF_CALCIUM_ION | 0.113273 | 0.001455 | 2.632655 | 0.011156 | 0.168038 | -2.94459 |
| GO_5_3_EXONUCLEASE_ACTIVITY | 0.162561 | -0.01346 | 2.624661 | 0.011389 | 0.169059 | -2.96238 |
| GO_MITOCHONDRIAL_GENE_EXPRESSION | 0.113842 | 0.012444 | 2.620868 | 0.011501 | 0.169059 | -2.97081 |
| GO_NADH_DEHYDROGENASE_COMPLEX_ASSEMBLY | 0.121446 | 0.007949 | 2.61663 | 0.011627 | 0.169059 | -2.98021 |
| GO_REGULATION_OF_MITOCHONDRIAL_FISSION | 0.138431 | 0.003502 | 2.61597 | 0.011647 | 0.169059 | -2.98167 |
| GO_REGULATION_OF_DIGESTIVE_SYSTEM_PROCESS | -0.10305 | 0.004221 | -2.61546 | 0.011663 | 0.169059 | -2.98281 |
| GO_PRIMARY_AMINO_COMPOUND_METABOLIC_PROCESS | -0.11953 | -0.00839 | -2.61533 | 0.011666 | 0.169059 | -2.98309 |
| GO_PHOSPHATE_ION_TRANSMEMBRANE_TRANSPORT | -0.19869 | -0.03408 | -2.61497 | 0.011677 | 0.169059 | -2.98389 |
| GO_TBP_CLASS_PROTEIN_BINDING | 0.117156 | 0.001452 | 2.614113 | 0.011703 | 0.169059 | -2.98579 |
| GO_U2_TYPE_CATALYTIC_STEP_2_SPLICEOSOME | 0.171991 | -0.00257 | 2.612002 | 0.011767 | 0.169544 | -2.99047 |
| GO_CYTOPLASMIC_PATTERN_RECOGNITION_RECEPTOR_SIGNALING_PATHWAY_IN_RESPONSE_TO_VIRUS | -0.12136 | 0.018129 | -2.61032 | 0.011818 | 0.169544 | -2.99419 |
| GO_NEGATIVE_REGULATION_OF_BLOOD_VESSEL_ENDOTHELIAL_CELL_MIGRATION | 0.115998 | 0.005629 | 2.610129 | 0.011824 | 0.169544 | -2.99462 |
| GO_THYMIC_T_CELL_SELECTION | 0.173812 | -0.0104 | 2.607554 | 0.011902 | 0.169544 | -3.00031 |
| GO_POSITIVE_REGULATION_OF_CHOLESTEROL_EFFLUX | -0.12471 | 0.026073 | -2.60683 | 0.011925 | 0.169544 | -3.00192 |
| GO_PEPTIDYL_SERINE_DEPHOSPHORYLATION | -0.11757 | 0.012412 | -2.6065 | 0.011935 | 0.169544 | -3.00264 |
| GO_REGULATION_OF_RYANODINE_SENSITIVE_CALCIUM_RELEASE_CHANNEL_ACTIVITY | 0.10968 | -0.00957 | 2.60419 | 0.012006 | 0.169995 | -3.00775 |
| GO_REGULATION_OF_TRANSLATIONAL_FIDELITY | 0.152497 | -0.00169 | 2.602932 | 0.012045 | 0.169995 | -3.01053 |
| GO_PARALLEL_FIBER_TO_PURKINJE_CELL_SYNAPSE | -0.14261 | 0.020114 | -2.60226 | 0.012066 | 0.169995 | -3.01202 |
| GO_ANTIGEN_PROCESSING_AND_PRESENTATION_OF_ENDOGENOUS_ANTIGEN | 0.130526 | -0.00461 | 2.60133 | 0.012094 | 0.169995 | -3.01407 |
| GO_GLYCOLIPID_CATABOLIC_PROCESS | 0.156327 | 0.006391 | 2.598832 | 0.012172 | 0.170099 | -3.01958 |
| GO_PHOSPHOLIPASE_A2_ACTIVITY_CONSUMING_1_2_DIPALMITOYLPHOSPHATIDYLCHOLINE | -0.10049 | 0.005461 | -2.59706 | 0.012228 | 0.17017 | -3.0235 |
| GO_INTERFERON_ALPHA_PRODUCTION | 0.121365 | -0.00019 | 2.595576 | 0.012274 | 0.17017 | -3.02676 |
| GO_FAT_SOLUBLE_VITAMIN_BIOSYNTHETIC_PROCESS | 0.134919 | 0.00426 | 2.591973 | 0.012388 | 0.17017 | -3.0347 |
| GO_POSITIVE_REGULATION_OF_T_CELL_RECEPTOR_SIGNALING_PATHWAY | 0.140191 | 0.019271 | 2.59113 | 0.012415 | 0.17017 | -3.03656 |
| GO_EXTRACELLULAR_TRANSPORT | -0.17051 | -0.00242 | -2.59058 | 0.012433 | 0.17017 | -3.03778 |
| GO_BUNDLE_OF_HIS_CELL_TO_PURKINJE_MYOCYTE_COMMUNICATION | -0.13292 | 0.00415 | -2.58795 | 0.012517 | 0.170867 | -3.04355 |
| GO_RESPONSE_TO_ACIDIC_PH | 0.12862 | -0.00122 | 2.582618 | 0.012689 | 0.172102 | -3.05527 |
| GO_RNA_POLYMERASE_III_COMPLEX | 0.149703 | 0.029846 | 2.575814 | 0.012911 | 0.174727 | -3.0702 |
| GO_EPITHELIAL_TO_MESENCHYMAL_TRANSITION_INVOLVED_IN_ENDOCARDIAL_CUSHION_FORMATION | 0.124125 | 0.001811 | 2.573628 | 0.012984 | 0.17531 | -3.07499 |
| GO_MODULATION_BY_VIRUS_OF_HOST_CELLULAR_PROCESS | -0.10179 | 0.004785 | -2.56768 | 0.013182 | 0.176531 | -3.088 |
| GO_PROTEIN_MANNOSYLATION | -0.10134 | 0.006849 | -2.56245 | 0.013359 | 0.177316 | -3.09943 |
| GO_MITOCHONDRIAL_RNA_PROCESSING | 0.140583 | 0.01596 | 2.55962 | 0.013456 | 0.178074 | -3.1056 |
| GO_MITOCHONDRIAL_TRNA_PROCESSING | 0.14674 | 0.024803 | 2.552253 | 0.01371 | 0.180248 | -3.12165 |
| GO_REGULATION_OF_B_CELL_RECEPTOR_SIGNALING_PATHWAY | 0.121219 | -0.00368 | 2.548421 | 0.013844 | 0.181613 | -3.12999 |
| GO_TRANSFORMING_GROWTH_FACTOR_BETA1_PRODUCTION | 0.14027 | 0.013764 | 2.544961 | 0.013966 | 0.182746 | -3.13751 |
| GO_POSITIVE_REGULATION_BY_HOST_OF_VIRAL_PROCESS | 0.129187 | -0.00241 | 2.542276 | 0.014062 | 0.183227 | -3.14333 |
| GO_RESPONSE_TO_INTERLEUKIN_12 | 0.107577 | 0.00854 | 2.539288 | 0.014169 | 0.183459 | -3.14981 |
| GO_RESPONSE_TO_FOLLICLE_STIMULATING_HORMONE | 0.128188 | 0.006903 | 2.534214 | 0.014352 | 0.184893 | -3.16081 |
| GO_RNA_POLYMERASE_ACTIVITY | 0.124293 | 0.017814 | 2.531943 | 0.014435 | 0.185301 | -3.16572 |
| GO_EUKARYOTIC_48S_PREINITIATION_COMPLEX | 0.141751 | -0.00661 | 2.529752 | 0.014515 | 0.185932 | -3.17046 |
| GO_INTRINSIC_COMPONENT_OF_PEROXISOMAL_MEMBRANE | -0.1182 | -0.01377 | -2.5287 | 0.014553 | 0.186027 | -3.17272 |
| GO_THYROID_HORMONE_METABOLIC_PROCESS | -0.11214 | 0.00306 | -2.52027 | 0.014866 | 0.188027 | -3.19092 |
| GO_REGULATION_OF_EXOSOMAL_SECRETION | -0.12802 | -0.00966 | -2.51739 | 0.014975 | 0.188485 | -3.19713 |
| GO_NEGATIVE_REGULATION_OF_SINGLE_STRANDED_VIRAL_RNA_REPLICATION_VIA_DOUBLE_STRANDED_DNA_INTERMEDIATE | 0.103787 | 0.004638 | 2.516803 | 0.014997 | 0.188485 | -3.19839 |
| GO_C_TERMINAL_PROTEIN_AMINO_ACID_MODIFICATION | 0.119435 | 0.010331 | 2.514403 | 0.015088 | 0.188835 | -3.20356 |
| GO_ORGANELLE_INHERITANCE | 0.113201 | 0.026069 | 2.510043 | 0.015254 | 0.189835 | -3.21293 |
| GO_PROTEIN_ADP_RIBOSYLATION | 0.101204 | 0.007956 | 2.509003 | 0.015294 | 0.189835 | -3.21517 |
| GO_SERINE_FAMILY_AMINO_ACID_BIOSYNTHETIC_PROCESS | -0.13657 | 0.010876 | -2.50714 | 0.015366 | 0.190122 | -3.21917 |
| GO_LOW_DENSITY_LIPOPROTEIN_PARTICLE_BINDING | 0.152546 | -0.0053 | 2.506757 | 0.015381 | 0.190122 | -3.21999 |
| GO_MYELOID_LEUKOCYTE_CYTOKINE_PRODUCTION | 0.138212 | -0.00285 | 2.505359 | 0.015435 | 0.190347 | -3.22299 |
| GO_LIVER_REGENERATION | 0.126739 | 0.016351 | 2.501776 | 0.015575 | 0.191333 | -3.23068 |
| GO_PROTEIN_LIPID_COMPLEX_ASSEMBLY | -0.11335 | 0.009315 | -2.50042 | 0.015628 | 0.19156 | -3.23357 |
| GO_REGULATION_OF_POSTSYNAPTIC_NEUROTRANSMITTER_RECEPTOR_ACTIVITY | -0.13036 | 0.017093 | -2.49792 | 0.015726 | 0.191623 | -3.23893 |
| GO_STRUCTURAL_CONSTITUENT_OF_RIBOSOME | 0.130965 | 0.008573 | 2.494274 | 0.015871 | 0.192991 | -3.24673 |
| GO_RNA_INTERFERENCE | 0.100012 | 0.002016 | 2.479151 | 0.016483 | 0.198421 | -3.27899 |
| GO_RETINAL_BINDING | 0.124823 | -0.00701 | 2.476725 | 0.016583 | 0.198617 | -3.28416 |
| GO_POSITIVE_REGULATION_OF_RHO_PROTEIN_SIGNAL_TRANSDUCTION | 0.124455 | -0.00358 | 2.475504 | 0.016633 | 0.198617 | -3.28675 |
| GO_RESPONSE_TO_PH | 0.108086 | 0.003281 | 2.474397 | 0.016679 | 0.198617 | -3.28911 |
| GO_AXONEMAL_DYNEIN_COMPLEX_ASSEMBLY | -0.18962 | -0.02358 | -2.47404 | 0.016694 | 0.198617 | -3.28986 |
| GO_NEGATIVE_REGULATION_OF_RNA_SPLICING | 0.155372 | 0.012346 | 2.473163 | 0.016731 | 0.198617 | -3.29173 |
| GO_NEGATIVE_REGULATION_OF_PEPTIDYL_THREONINE_PHOSPHORYLATION | 0.112587 | 0.01273 | 2.470895 | 0.016826 | 0.198987 | -3.29654 |
| GO_BLASTOCYST_GROWTH | 0.111115 | -0.0034 | 2.470832 | 0.016828 | 0.198987 | -3.29668 |
| GO_RNA_POLYMERASE_II_CORE_COMPLEX | 0.138652 | 0.017494 | 2.467118 | 0.016985 | 0.199346 | -3.30456 |
| GO_POSITIVE_THYMIC_T_CELL_SELECTION | 0.186669 | -0.00149 | 2.466507 | 0.017011 | 0.199346 | -3.30585 |
| GO_PROTEIN_AUTO_ADP_RIBOSYLATION | 0.142003 | 0.023922 | 2.459462 | 0.017311 | 0.201261 | -3.32077 |
| GO_REGULATION_OF_CELL_PROJECTION_SIZE | -0.13554 | 0.011824 | -2.45725 | 0.017407 | 0.201847 | -3.32545 |
| GO_RIBOSOMAL_SUBUNIT | 0.122449 | 0.005874 | 2.452198 | 0.017627 | 0.203606 | -3.33612 |
| GO_MATURATION_OF_LSU_RRNA | 0.145868 | -0.00033 | 2.450287 | 0.01771 | 0.20418 | -3.34015 |
| GO_CALCIUM_MEDIATED_SIGNALING_USING_INTRACELLULAR_CALCIUM_SOURCE | 0.114263 | -0.00566 | 2.446602 | 0.017873 | 0.205614 | -3.34791 |
| GO_MITOCHONDRIAL_SMALL_RIBOSOMAL_SUBUNIT | 0.14351 | 0.005911 | 2.444399 | 0.017971 | 0.205614 | -3.35255 |
| GO_U2_TYPE_SPLICEOSOMAL_COMPLEX | 0.117272 | -0.01378 | 2.444372 | 0.017972 | 0.205614 | -3.35261 |
| GO_BLOC_1_COMPLEX | 0.112202 | -0.00495 | 2.437255 | 0.018291 | 0.208472 | -3.36757 |
| GO_COTRANSLATIONAL_PROTEIN_TARGETING_TO_MEMBRANE | 0.131323 | 0.022436 | 2.436295 | 0.018335 | 0.20857 | -3.36959 |
| GO_GLUTATHIONE_DERIVATIVE_METABOLIC_PROCESS | 0.135018 | 0.002241 | 2.434256 | 0.018427 | 0.208642 | -3.37387 |
| GO_OXIDOREDUCTASE_ACTIVITY_ACTING_ON_THE_CH_NH2_GROUP_OF_DONORS_OXYGEN_AS_ACCEPTOR | 0.101256 | -0.01948 | 2.432177 | 0.018522 | 0.209116 | -3.37823 |
| GO_POSITIVE_REGULATION_OF_MACROPHAGE_ACTIVATION | 0.134585 | -0.00244 | 2.429246 | 0.018657 | 0.210238 | -3.38437 |
| GO_GLUCOCORTICOID_BIOSYNTHETIC_PROCESS | -0.14239 | -0.00431 | -2.42823 | 0.018704 | 0.210368 | -3.38651 |
| GO_NEGATIVE_REGULATION_OF_MEIOTIC_NUCLEAR_DIVISION | 0.156946 | 0.018969 | 2.426422 | 0.018787 | 0.210401 | -3.39029 |
| GO_LIPID_DROPLET_ORGANIZATION | 0.101692 | 0.017453 | 2.425125 | 0.018847 | 0.210409 | -3.393 |
| GO_EXTRACELLULAR_VESICLE_BIOGENESIS | -0.10763 | 0.005279 | -2.42312 | 0.018941 | 0.210964 | -3.39719 |
| GO_RESPIRATORY_ELECTRON_TRANSPORT_CHAIN | 0.101502 | 0.005596 | 2.422547 | 0.018967 | 0.210964 | -3.39839 |
| GO_NEGATIVE_REGULATION_OF_SPROUTING_ANGIOGENESIS | 0.103337 | -0.00343 | 2.418701 | 0.019148 | 0.212064 | -3.40643 |
| GO_PROTEIN_HORMONE_RECEPTOR_ACTIVITY | -0.13795 | 0.003095 | -2.41721 | 0.019218 | 0.212064 | -3.40953 |
| GO_LYMPHOCYTE_COSTIMULATION | 0.104773 | -0.0024 | 2.416231 | 0.019265 | 0.212064 | -3.41158 |
| GO_CORNIFIED_ENVELOPE | -0.12564 | -0.01865 | -2.41524 | 0.019312 | 0.212064 | -3.41365 |
| GO_STRUCTURAL_CONSTITUENT_OF_POSTSYNAPSE | -0.13176 | -0.00881 | -2.41465 | 0.019339 | 0.212064 | -3.41487 |
| GO_GLUCOSIDASE_ACTIVITY | 0.125453 | 0.023302 | 2.414542 | 0.019345 | 0.212064 | -3.4151 |
| GO_COPII_VESICLE_COAT | 0.14039 | 0.003214 | 2.412994 | 0.019418 | 0.212434 | -3.41833 |
| GO_INSULIN_LIKE_GROWTH_FACTOR_RECEPTOR_BINDING | 0.114125 | -0.00646 | 2.410541 | 0.019536 | 0.213329 | -3.42344 |
| GO_PROTEIN_MEMBRANE_ADAPTOR | -0.13971 | 0.012892 | -2.4086 | 0.019629 | 0.213501 | -3.42748 |
| GO_DIHYDROLIPOYL_DEHYDROGENASE_COMPLEX | -0.14686 | -0.00162 | -2.40766 | 0.019675 | 0.213501 | -3.42943 |
| GO_VENOUS_BLOOD_VESSEL_DEVELOPMENT | 0.150568 | 0.010687 | 2.405167 | 0.019795 | 0.213501 | -3.43462 |
| GO_PCG_PROTEIN_COMPLEX | 0.118382 | -0.00615 | 2.403001 | 0.019901 | 0.214192 | -3.43912 |
| GO_RIG_I_SIGNALING_PATHWAY | -0.13099 | 0.008002 | -2.39314 | 0.020387 | 0.217864 | -3.45958 |
| GO_ENDOSOME_LUMEN | 0.120674 | 0.001469 | 2.38757 | 0.020666 | 0.219672 | -3.47109 |
| GO_ANKYRIN_BINDING | -0.10053 | 0.005372 | -2.38724 | 0.020683 | 0.219672 | -3.47177 |
| GO_NEGATIVE_REGULATION_OF_VASCULAR_SMOOTH_MUSCLE_CELL_PROLIFERATION | 0.157337 | 0.00133 | 2.386846 | 0.020703 | 0.219672 | -3.47258 |
| GO_AEROBIC_ELECTRON_TRANSPORT_CHAIN | 0.123998 | 0.011797 | 2.376287 | 0.021243 | 0.223817 | -3.49436 |
| GO_DETOXIFICATION_OF_INORGANIC_COMPOUND | -0.16831 | 0.007662 | -2.37388 | 0.021368 | 0.224344 | -3.49932 |
| GO_CIS_TRANS_ISOMERASE_ACTIVITY | 0.117441 | -0.00189 | 2.372339 | 0.021448 | 0.224439 | -3.50248 |
| GO_NEGATIVE_REGULATION_OF_UBIQUITIN_PROTEIN_TRANSFERASE_ACTIVITY | 0.14176 | 0.021817 | 2.372268 | 0.021452 | 0.224439 | -3.50263 |
| GO_PERICARDIUM_DEVELOPMENT | 0.112476 | -0.01226 | 2.370923 | 0.021522 | 0.224782 | -3.50539 |
| GO_POSITIVE_REGULATION_OF_FERTILIZATION | -0.11729 | -0.01391 | -2.36874 | 0.021637 | 0.225018 | -3.50989 |
| GO_SODIUM_TRANSMEMBRANE_TRANSPORTER_ACTIVITY_PHOSPHORYLATIVE_MECHANISM | -0.11732 | -0.005 | -2.3684 | 0.021654 | 0.225018 | -3.51057 |
| GO_TRICARBOXYLIC_ACID_CYCLE | 0.10352 | 0.021204 | 2.368347 | 0.021657 | 0.225018 | -3.51069 |
| GO_REGULATION_OF_FATTY_ACID_TRANSPORT | -0.10986 | 0.007306 | -2.36521 | 0.021823 | 0.22609 | -3.51713 |
| GO_REGULATION_OF_ARP2_3_COMPLEX_MEDIATED_ACTIN_NUCLEATION | -0.10036 | 0.019726 | -2.35869 | 0.022171 | 0.227382 | -3.53047 |
| KEGG_ALPHA_LINOLENIC_ACID_METABOLISM | -0.12312 | -0.0076 | -2.66851 | 0.010068 | 0.228428 | -2.76956 |
| GO_PEPTIDE_ANTIGEN_BINDING | 0.154715 | 0.011288 | 2.350631 | 0.022608 | 0.23011 | -3.54695 |
| GO_NEGATIVE_REGULATION_OF_AXON_GUIDANCE | 0.107226 | -0.02975 | 2.347488 | 0.02278 | 0.231061 | -3.55337 |
| GO_COA_HYDROLASE_ACTIVITY | 0.121139 | 0.014165 | 2.346824 | 0.022817 | 0.231061 | -3.55472 |
| GO_ATP_SYNTHESIS_COUPLED_ELECTRON_TRANSPORT | 0.107932 | 0.004426 | 2.34151 | 0.023112 | 0.232866 | -3.56554 |
| GO_NEGATIVE_REGULATION_OF_DENDRITE_MORPHOGENESIS | -0.10958 | 0.017969 | -2.33231 | 0.02363 | 0.236075 | -3.58423 |
| GO_SINGLE_STRANDED_VIRAL_RNA_REPLICATION_VIA_DOUBLE_STRANDED_DNA_INTERMEDIATE | 0.102525 | -0.00094 | 2.332204 | 0.023636 | 0.236075 | -3.58445 |
| GO_NOREPINEPHRINE_SECRETION | -0.11054 | -0.00659 | -2.33207 | 0.023644 | 0.236075 | -3.58472 |
| GO_RIBOSOME | 0.107408 | 0.006326 | 2.329351 | 0.023799 | 0.236496 | -3.59023 |
| GO_CELLULAR_RESPONSE_TO_GONADOTROPIN_STIMULUS | 0.101227 | 0.000267 | 2.328861 | 0.023827 | 0.236496 | -3.59123 |
| GO_DISRUPTION_OF_CELLS_OF_OTHER_ORGANISM_INVOLVED_IN_SYMBIOTIC_INTERACTION | -0.15322 | 0.02223 | -2.3247 | 0.024067 | 0.238089 | -3.59966 |
| GO_ORGANOPHOSPHATE_ESTER_TRANSMEMBRANE_TRANSPORTER_ACTIVITY | -0.10131 | 0.007095 | -2.32205 | 0.024221 | 0.239191 | -3.60502 |
| GO_REGULATION_OF_TRIGLYCERIDE_CATABOLIC_PROCESS | -0.11257 | 0.010682 | -2.31605 | 0.024572 | 0.239191 | -3.61713 |
| GO_PROTON_TRANSPORTING_TWO_SECTOR_ATPASE_COMPLEX_PROTON_TRANSPORTING_DOMAIN | 0.130374 | 0.017105 | 2.315953 | 0.024577 | 0.239191 | -3.61732 |
| GO_ESTABLISHMENT_OR_MAINTENANCE_OF_ACTIN_CYTOSKELETON_POLARITY | 0.106588 | 0.002168 | 2.314215 | 0.02468 | 0.239225 | -3.62082 |
| GO_REGULATION_OF_NEUTROPHIL_MIGRATION | 0.110414 | -0.00162 | 2.313868 | 0.024701 | 0.239225 | -3.62152 |
| GO_POSITIVE_REGULATION_OF_DOUBLE_STRAND_BREAK_REPAIR_VIA_NONHOMOLOGOUS_END_JOINING | 0.140247 | 0.035904 | 2.310882 | 0.024878 | 0.23978 | -3.62754 |
| GO_COPII_COATED_VESICLE_CARGO_LOADING | 0.129871 | 0.000283 | 2.310084 | 0.024925 | 0.239853 | -3.62915 |
| GO_STAGA_COMPLEX | 0.121166 | -0.00124 | 2.308664 | 0.02501 | 0.240284 | -3.632 |
| GO_EUKARYOTIC_TRANSLATION_INITIATION_FACTOR_3_COMPLEX | 0.131405 | 0.011112 | 2.305139 | 0.025222 | 0.241497 | -3.63909 |
| GO_VASCULAR_ENDOTHELIAL_GROWTH_FACTOR_PRODUCTION | 0.104708 | -0.00505 | 2.301994 | 0.025413 | 0.241497 | -3.64541 |
| GO_NUCLEOSIDE_MONOPHOSPHATE_BIOSYNTHETIC_PROCESS | 0.101272 | 0.012451 | 2.301893 | 0.025419 | 0.241497 | -3.64561 |
| GO_MHC_PROTEIN_COMPLEX_BINDING | 0.118516 | 0.003362 | 2.30011 | 0.025527 | 0.241761 | -3.64919 |
| GO_NEURAL_CREST_FORMATION | 0.118434 | 0.005467 | 2.294097 | 0.025896 | 0.243231 | -3.66124 |
| GO_ALCOHOL_DEHYDROGENASE_NADPPLUS_ACTIVITY | 0.13222 | -0.01227 | 2.294007 | 0.025902 | 0.243231 | -3.66142 |
| GO_GLYCINE_BINDING | -0.12851 | -0.01589 | -2.29344 | 0.025937 | 0.243231 | -3.66255 |
| GO_PURINE_NUCLEOBASE_BIOSYNTHETIC_PROCESS | 0.157481 | 0.006801 | 2.29296 | 0.025966 | 0.243231 | -3.66351 |
| GO_PEPTIDASE_ACTIVATOR_ACTIVITY_INVOLVED_IN_APOPTOTIC_PROCESS | 0.111456 | 0.011551 | 2.289894 | 0.026157 | 0.244251 | -3.66965 |
| KEGG_GLYCOSPHINGOLIPID_BIOSYNTHESIS_GANGLIO_SERIES | 0.165042 | -0.00538 | 2.491119 | 0.015876 | 0.24636 | -3.1513 |
| KEGG_GLUTATHIONE_METABOLISM | 0.103896 | 0.000371 | 2.447766 | 0.017694 | 0.24636 | -3.24152 |
| GO_CELLULAR_RESPONSE_TO_COPPER_ION | -0.16848 | -0.00578 | -2.27857 | 0.026871 | 0.248294 | -3.69224 |
| GO_RNAI_EFFECTOR_COMPLEX | -0.11587 | -0.01224 | -2.27843 | 0.02688 | 0.248294 | -3.69252 |
| GO_OXALATE_TRANSPORT | -0.14654 | 0.021068 | -2.27654 | 0.027001 | 0.248647 | -3.69628 |
