# Supplementary material for: Three Oxidative Stress-Related Genes That Associate Endometrial Immune Cells Are Considered as Potential Biomarkers for the Prediction of Unexplained Recurrent Implantation Failure
Source: Front Immunol. 2022 Jun 3;13:902268. doi: 10.3389/fimmu.2022.902268 (PMC9203891; doi:10.3389/fimmu.2022.902268)
Supplement: Supplementary file 2 [file Table_2.docx]

Table S2 ORGs extracted from the GO_ OXIDATIVE_STRESS gene set in Molecular Signatures Database

| AKT1 |
| --- |
| ARL6IP5 |
| BAG5 |
| BCL2 |
| CYP1B1 |
| DIABLO |
| FBXW7 |
| GPX1 |
| GSKIP |
| HIF1A |
| HSPB1 |
| HTRA2 |
| INS |
| JAK2 |
| MAP3K5 |
| MAPK7 |
| MCL1 |
| MELK |
| NFE2L2 |
| NME5 |
| NOL3 |
| NONO |
| NOX1 |
| P4HB |
| PARK7 |
| PARP1 |
| PDCD10 |
| PDK1 |
| PINK1 |
| PML |
| PRKCD |
| PRKN |
| PRODH |
| SFPQ |
| SIRT1 |
| SOD1 |
| SOD2 |
| STK24 |
| STK25 |
| TRAP1 |
| UBQLN1 |
| VNN1 |
| ZNF622 |
| ATF4 |
| ATG7 |
| CTNNB1 |
| FBXO7 |
| FYN |
| FZD1 |
| GATA4 |
| GGT7 |
| GPR37 |
| GPR37L1 |
| HGF |
| IL10 |
| LRRK2 |
| MCTP1 |
| MEAK7 |
| MET |
| MIR132 |
| MIR133A1 |
| MIR133A2 |
| MIR17 |
| MIR195 |
| MIR19A |
| MIR21 |
| MIR29B1 |
| MIR29B2 |
| MIR92A1 |
| MIR92A2 |
| MIRLET7B |
| NCOA7 |
| NR4A3 |
| OXR1 |
| PAGE4 |
| PDE8A |
| PSAP |
| PYCR1 |
| RACK1 |
| REST |
| SLC7A11 |
| TSC1 |
| TXN |
| WNT1 |
| ENDOG |
| HDAC6 |
| MIR34A |
| MMP3 |
| PAWR |
| RIPK1 |
| TLR4 |
| TLR6 |
| ACOX2 |
| RGN |
| SELENON |
| TNF |
| CHD6 |
| MT3 |
| SESN2 |
| LANCL1 |
| STK26 |
| TP53 |
| ABCD1 |
| BMP7 |
| CD36 |
| DHFR |
| DHFRP1 |
| FBLN5 |
| FUT8 |
| GCH1 |
| MACROH2A1 |
| MAPKAP1 |
| SESN1 |
| SESN3 |
| STOX1 |
| SZT2 |
| ANKRD2 |
| ARNT |
| EPAS1 |
| HMOX1 |
| SIN3A |
| ABCC2 |
| ABL1 |
| ADA |
| ADAM9 |
| ADIPOQ |
| ADNP2 |
| ADPRHL2 |
| AGAP3 |
| AIF1 |
| AIFM1 |
| AKR1C3 |
| ALAD |
| ALDH3B1 |
| ALS2 |
| ANGPTL7 |
| ANKZF1 |
| ANXA1 |
| APEX1 |
| APOA4 |
| APOD |
| APOE |
| APP |
| APTX |
| AQP1 |
| AREG |
| ARG1 |
| ARNTL |
| ATOX1 |
| ATP13A2 |
| ATP2A2 |
| ATP7A |
| ATRN |
| AXL |
| BAD |
| BAK1 |
| BECN1 |
| BNIP3 |
| BRF2 |
| BTG1 |
| BTK |
| C19orf12 |
| CA3 |
| CAMKK2 |
| CAPN2 |
| CASP3 |
| CAT |
| CBX8 |
| CCL19 |
| CCNA2 |
| CCR7 |
| CCS |
| CD38 |
| CDK1 |
| CDK2 |
| CFLAR |
| CHRNA4 |
| CHUK |
| CLN8 |
| COA8 |
| COL1A1 |
| CPEB2 |
| CRK |
| CRYAB |
| CRYGD |
| CYBA |
| CYBB |
| CYCS |
| CYGB |
| CYP2E1 |
| DAPK1 |
| DGKK |
| DHCR24 |
| DHRS2 |
| DNM2 |
| DPEP1 |
| DUOX1 |
| DUOX2 |
| DUSP1 |
| ECT2 |
| EDN1 |
| EEF2 |
| EGFR |
| EGLN1 |
| EIF2S1 |
| EPX |
| ERCC1 |
| ERCC2 |
| ERCC3 |
| ERCC6 |
| ERCC6L2 |
| ERCC8 |
| ERO1A |
| ETFDH |
| ETS1 |
| ETV5 |
| EZH2 |
| FABP1 |
| FANCC |
| FANCD2 |
| FER |
| FGF8 |
| FKBP1B |
| FOS |
| FOSL1 |
| FOXO1 |
| FOXO3 |
| FXN |
| G6PD |
| GCLC |
| GCLM |
| GJB2 |
| GLRX2 |
| GNAO1 |
| GPX2 |
| GPX3 |
| GPX4 |
| GPX5 |
| GPX6 |
| GPX7 |
| GPX8 |
| GSR |
| GSS |
| GSTP1 |
| GUCY1B1 |
| HAO1 |
| HBA1 |
| HBA2 |
| HBB |
| HDAC2 |
| HMOX2 |
| HNRNPD |
| HNRNPM |
| HP |
| HSF1 |
| HSPA1A |
| HSPA1B |
| HYAL1 |
| HYAL2 |
| IDH1 |
| IL18RAP |
| IL6 |
| IMPACT |
| IPCEF1 |
| JUN |
| KCNA5 |
| KCNC2 |
| KDM6B |
| KEAP1 |
| KLF2 |
| KLF4 |
| KPNA4 |
| KRT1 |
| LCN2 |
| LDHA |
| LIAS |
| LONP1 |
| LPO |
| MAP1LC3A |
| MAPK1 |
| MAPK13 |
| MAPK3 |
| MAPK8 |
| MAPK9 |
| MAPT |
| MB |
| MBL2 |
| MDM2 |
| MGAT3 |
| MGST1 |
| MICB |
| MIR103A1 |
| MIR103A2 |
| MIR107 |
| MMP14 |
| MMP2 |
| MMP9 |
| MPO |
| MPV17 |
| MSRA |
| MSRB2 |
| MSRB3 |
| MT-CO1 |
| MT-ND1 |
| MT-ND3 |
| MT-ND5 |
| MT-ND6 |
| MTF1 |
| MTR |
| MYB |
| MYEF2 |
| NAPRT |
| NCF1 |
| NCF2 |
| NCF4 |
| NDUFA12 |
| NDUFA6 |
| NDUFB4 |
| NDUFS2 |
| NDUFS8 |
| NEIL1 |
| NET1 |
| NFE2L1 |
| NME2 |
| NME8 |
| NOS3 |
| NOX4 |
| NOX5 |
| NQO1 |
| NR4A2 |
| NUDT1 |
| NUDT15 |
| NUDT2 |
| OGG1 |
| OSER1 |
| OXSR1 |
| PAX2 |
| PCGF2 |
| PCNA |
| PDGFD |
| PDGFRA |
| PDGFRB |
| PDK2 |
| PDLIM1 |
| PENK |
| PKD2 |
| PLA2R1 |
| PLEKHA1 |
| PLK3 |
| PNKP |
| PNPT1 |
| PON2 |
| PPARGC1A |
| PPARGC1B |
| PPIF |
| PPP1R15B |
| PPP2CB |
| PPP5C |
| PRDX1 |
| PRDX2 |
| PRDX3 |
| PRDX4 |
| PRDX5 |
| PRDX6 |
| PRKAA1 |
| PRKAA2 |
| PRKD1 |
| PRKRA |
| PRNP |
| PRR5L |
| PSEN1 |
| PSIP1 |
| PSMB5 |
| PTGS1 |
| PTGS2 |
| PTK2B |
| PTPRK |
| PTPRN |
| PXDN |
| PXDNL |
| PXN |
| PYCR2 |
| PYROXD1 |
| RAD52 |
| RBM11 |
| RBPMS |
| RELA |
| RGS14 |
| RHOB |
| RIPK3 |
| RNF112 |
| ROMO1 |
| RPS3 |
| RRM2B |
| RWDD1 |
| S100A7 |
| SCARA3 |
| SCGB1A1 |
| SDC1 |
| SELENOK |
| SELENOP |
| SELENOS |
| SETX |
| SGK2 |
| SIGMAR1 |
| SIRPA |
| SIRT2 |
| SLC23A2 |
| SLC25A24 |
| SLC8A1 |
| SMPD3 |
| SNCA |
| SOD3 |
| SP1 |
| SRC |
| SRXN1 |
| STAR |
| STAT1 |
| STAT6 |
| STAU1 |
| STC2 |
| STX2 |
| STX4 |
| TAT |
| TBC1D24 |
| TMEM161A |
| TNFAIP3 |
| TOR1A |
| TP53INP1 |
| TPM1 |
| TPO |
| TRAF2 |
| TREX1 |
| TRPA1 |
| TRPC6 |
| TRPM2 |
| TXN2 |
| TXNIP |
| TXNRD1 |
| TXNRD2 |
| UBE3A |
| UCN |
| UCP1 |
| UCP3 |
| VKORC1L1 |
| VRK2 |
| WNT16 |
| WRN |
| XPA |
| XRCC1 |
| ZC3H12A |
| ZNF277 |
| ZNF580 |
